# Development of a Validated Lay Checklist (Info Without Side Effects) for Assessing Health Information on Websites: Mixed Methods Study

**DOI:** 10.2196/69529

**Published:** 2026-05-20

**Authors:** Ursula Griebler, Irma Klerings, Christina Koscher-Kien, Benedikt Lutz, Eva Krczal, Dominic Ledinger, Iris Mair, Robert Emprechtinger, Filiz Keser Aschenberger, Bernd Kerschner

**Affiliations:** 1 Department for Evidence-based Medicine and Evaluation, University for Continuing Education Krems, 3500 Krems an der Donau, Austria, 43 2732893 ext 2914; 2 Department for Knowledge and Communication Management, University for Continuing Education Krems, Krems an der Donau, Austria; 3 Department for Economy and Health, University for Continuing Education Krems, Krems an der Donau, Austria; 4 QUEST Center for Responsible Research, Berlin Institute of Health at Charité (BIH), Berlin, Germany; 5 Department for Continuing Education Research and Educational Technologies, University for Continuing Education Krems, Krems an der Donau, Austria

**Keywords:** health information, online health information, health information websites, checklist, critical health literacy

## Abstract

**Background:**

The internet has become a major source of health information; yet, the quality of health information on websites varies considerably. Users’ ability to evaluate either the factual accuracy or the trustworthiness of health information on websites is limited, as around half of the European people have limited health literacy. Existing checklists and tools are either prepared for research purposes or to be used by health care professionals. They do not account for the lay user perspective, since they are too long and complicated to be used by laypersons, or were developed for printed health information only.

**Objective:**

The aim of the study was to develop and validate a checklist that enables laypersons to evaluate the trustworthiness of health information on websites without requiring prior training.

**Methods:**

We used a multistage mixed methods approach including (1) a comprehensive literature review to identify existing tools and quality criteria, (2) an expert Delphi study with 6 specialists in patient communication and health information, (3) 2 rounds of cognitive interviews with 19 lay users, (4) application testing on 15 selected web pages with information about health interventions with 20 additional lay users, (5) a determination of the factual correctness of 100 web pages with health information by assessing the difference between the claimed and factual strength of the evidence on these web pages, and (6) validation testing by research team members on these 100 web pages using a Bayesian logistic regression model to analyze the predictive validity. In the final step, we integrated all quantitative and qualitative results to select the final checklist items.

**Results:**

From an initial pool of 1740 items extracted from 73 documents, we systematically reduced the list through multiple evaluation and testing rounds. To ensure the checklist is user-friendly, we involved a diverse group of potential users. The final product, the Info Without Side Effects (iWISE) checklist, contains seven items that assess key aspects of health information trustworthiness, including the absence of advertising, balanced presentation of information, the limited use of professional jargon, origination from an independent organization, citation of sources, mention of scientific validation, and the presence of a publication date. The checklist demonstrated the ability to distinguish between evidence-based and nonevidence-based health information web pages in the German language: the validation testing showed that when all the items were marked with yes, there was a nearly 100% probability that the health information was also factually correct.

**Conclusions:**

The iWISE checklist represents a user-friendly, validated tool for evaluating the trustworthiness of health information about interventions on websites. With only 7 items, it is easy to remember and could significantly improve critical health literacy. Future research should test its reliability for social media posts and health information videos.

## Introduction

With its accessibility and vast resources, the internet has become an increasingly popular source of health information for the general public. Individuals frequently turn to online platforms to seek answers to their health-related questions, to research symptoms, and to explore treatment options. A recent survey in the European Union reported that in 2024, 58% of individuals aged 16 to 74 years used the internet for health-related information-seeking, with the highest rates up to 82% in the Netherlands, 79% in Finland, and 78% in Denmark. In Austria, 65% used the internet to search for information on health issues [1].

While the abundance of health information available online can be empowering, it also presents significant challenges. The quality and reliability of this information vary widely, ranging from evidence-based medical advice to potentially harmful misinformation [2,3]. This disparity raises concerns about lay users’ ability to discern trustworthy sources from unreliable ones. The latest European health literacy survey showed that more than half of users have difficulties in deciding whether the information they find online is trustworthy and objective [4]. According to a survey by the Bertelsmann Foundation [5], between 64% and 84% of participants in Germany rely on advertisement-funded or scientifically unverified web content. Available evidence-based websites were known to only 18% to 27% of respondents; yet, the majority surprisingly judged such evidence-based websites as untrustworthy [5]. The ability to judge the trustworthiness of health information depends on an adequate level of health literacy. Yet, the latest Health Literacy Survey in Europe in 2020 showed that at least 1 of 10 (12.4%) participants had inadequate health literacy, and almost every second respondent (47.6%) in the total sample had limited (inadequate or problematic) health literacy, with substantial differences between member states [4].

Health literacy is a multifaceted construct that “entails people’s knowledge, motivation, and competences to access, understand, appraise, and apply health information in order to make judgments and take decisions in everyday life concerning health care, disease prevention, and health promotion” [6].

Low health literacy is known to negatively affect health [7]; it leads to poor health decisions and inferior treatment outcomes as well as to higher hospitalization, morbidity, and premature death rates [6]. Low health literacy may also lead to disadvantageous consumer choices when confronted with exaggerated claims about health services, over-the-counter medications, or nutritional supplements. It has been shown that people with lower health literacy have greater difficulty evaluating and differentiating low-quality from high-quality health information [8].

Strengthening health literacy is also an important factor for achieving the United Nations’ Sustainable Development Goal Number 3: ensuring healthy lives and promoting well-being for all at all ages [9].

Critical health literacy, an essential aspect of health literacy, can play a crucial role in addressing these issues. Critical health literacy is a higher-order cognitive process that can be defined as “the ability to access, understand, and manage health information as well as the ability to assess its credibility and to critically analyze and, where appropriate, challenge the information” [10]. It leads to individual and community empowerment by raising critical consciousness, improving quality of life and health behavior, and enhancing health-related social and political action [10]. One way to enhance critical health literacy involves using a tool for assessing the reliability of health information. To be useful, a tool must be based on operationalized quality criteria, should be validated, and must be easily understood and applied by laypersons [11,12]. According to a 2020 systematic review [12], none of the many preexisting checklist tools fulfill all these requirements. This is also true for the recently developed Mapping the Quality of Health Information (MAPPinfo) checklist [11], a German-language tool that was later translated into English. However, untrained laypersons may find it too complex to apply in their daily routines: the 19-item tool is supplemented by 13 pages of explanations in its German version [13] and 12 pages in its English [14]. It was validated based on operationalized and evidence-based criteria only by nursing and medical students [11].

The aim of our study was, therefore, to develop a validated checklist for laypersons to evaluate the trustworthiness of health information on websites without prior training and with minimal necessary explanations. Similar to MAPPinfo, we developed and validated our tool for a German-speaking audience and present here an English-language translation.

## Methods

### Working Definitions

#### Health Information on Websites

We defined health information on websites as any health-related content accessible on a web page via an online search engine (eg, Google). A website consists of multiple web pages under one domain. In this study, we focused on information about health interventions, defined as treatments claimed or believed to relieve or prevent symptoms or diseases (eg, vitamin C for the treatment of a cold). Health information from social media platforms or artificial intelligence–generated summaries was not included.

#### Trustworthiness of Health Information on Websites

For the purpose of this study, we defined trustworthiness of health information on websites as the degree to which the information on a given web page can be trusted by laypersons to be reliable and valid. This definition aligns with Viviani and Pasi’s [15] conceptualization of trustworthiness as a key dimension of credibility, focusing specifically on the audience’s perception. As laypersons generally do not have the domain expertise to directly judge whether health information is factually correct, we use trustworthiness as a proxy for the correctness, that is, the accuracy of the information.

#### Laypersons

Following the Cambridge Dictionary [16], we defined layperson as someone who is not an expert in or does not have detailed knowledge of health-related topics, that is, persons without a formal health-related education (ie, no medical doctors, nurses, or other health care professionals or health researchers).

### Study Design

**Figure 1.**
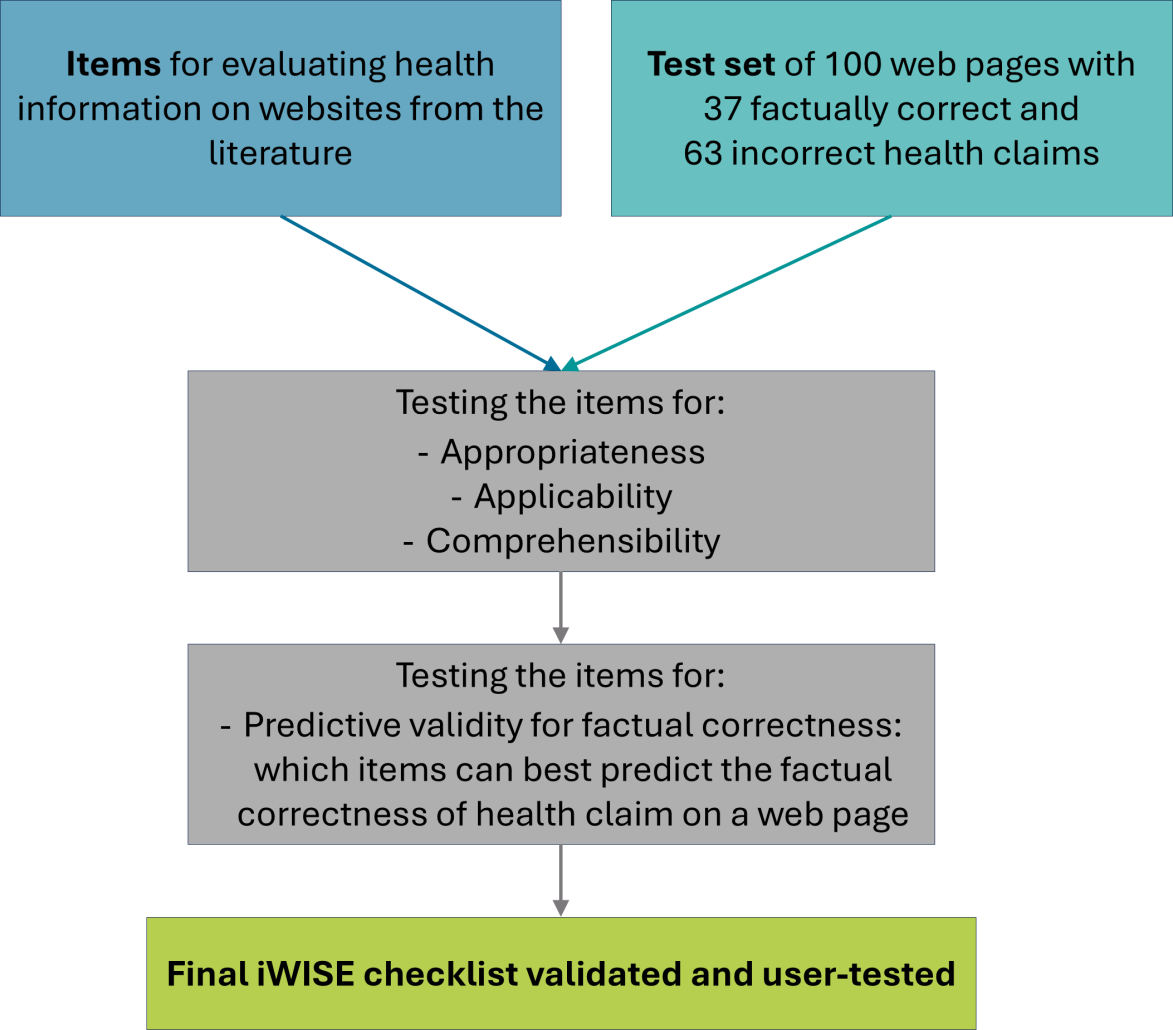
Overview of the development process of the Info Without Side Effects (iWISE) checklist.

The Info Without Side Effects (iWISE) checklist was developed using a sequential, multistage mixed methods design, in which the findings from each step iteratively informed focus and data collection of subsequent stages [17]. The development process comprised five steps: (1) a comprehensive literature review, (2) an expert Delphi study, (3) cognitive interviews with lay users, (4) application tests with laypersons, and (5) predictive validation with a test set (see Figure 1 for a short overview of the development process). For a detailed description of the process, please see the Detailed Methods Description section in Multimedia Appendix 1.

We developed a protocol a priori and published and registered it in the Open Science Framework retrospectively [18]. During the study, we made a few amendments to the protocol.

### Ethical Considerations

The study followed the University for Continuing Education Krems’ privacy policy (Article 13 General Data Protection Regulation) and was approved by the university’s ethics commission (EK GZ 23/2021‐2024). Interview participants received written study information and provided verbal informed consent; they received approximately US $35 voucher as compensation. Transcripts were deidentified by replacing all names and other identifiable details with alphanumeric codes. To ensure comprehensive reporting, we adhered to the Standards for Reporting Qualitative Research (SRQR) [19] and the American Psychological Association Style Journal Article Reporting Standards for mixed methods research [20].

### Literature Search and Selection

We conducted a tailored literature search [21] led by an experienced information specialist (IK) to identify existing checklists and tools as well as conceptual literature relevant to evaluate (online) health information. The iterative search was conducted between March and September 2021 and combined a variety of search methods:

- Database searches in PubMed, Scopus, Epistemonikos, and Library, Information Science & Technology Abstracts (searched via EBSCO) to retrieve reviews about the evaluation of health information.
- General web searches via Google, Google Scholar, and the Bielefeld Academic Search Engine to identify further published and gray literature.
- Reference list checking of all the (systematic) reviews identified throughout the search process.

One person screened all the results, and a second person verified the inclusion or exclusion decision. From each included (systematic) review on tools and checklists, we extracted the tools’ and checklists’ names and references.

### Data Extraction and Categorization of the Items

#### Data Extraction

In total, 7 researchers (BK, CK-K, IK, EK, UG, FKA, and Isolde Sommer) extracted the data into a structured matrix in Microsoft Excel. The matrix captured (1) general information (eg, reference type, tool or checklist name, target group, application field, and validation status) and (2) domains, subdomains, item texts, and answer categories or descriptions. For the tools or checklists identified in the (systematic) reviews, we extracted the included names and references of the tool or checklist and added the most frequently mentioned tools or checklists that we had not included yet.

#### Categorization of the Items

In total, 4 members of the research team (CK-K, EK, IK, and UG) categorized all the extracted items into 5 content categories and adjacent subcategories using an iteratively developed structure (Table S1 in Multimedia Appendix 1). We excluded criteria deemed unsuitable, based on the adapted feasibility criteria by Provost et al [22]: timeliness, expertise independence, externability, generalizability, scope of our project, category without items, unclear wording, and open questions. A second person checked the categorization and exclusions; uncertainties were discussed and resolved among the team.

### Item Reduction

First, 4 team researchers (CK-K, EK, IK, and UG) merged duplicate and similarly worded items and translated the English items into German. We standardized the item wording by phrasing all items as questions and aligning the responses so that “yes” indicated trustworthiness. The process was done by one person and checked by a second person, and discrepancies or conflicts were resolved by discussion.

Second, we rated each item’s appropriateness (relevance for assessing trustworthiness) and applicability (ease of use without prior knowledge) on 5-point scales in an Excel matrix. In total, 8 researchers (EK, FKA, BL, UG, BK, CK-K, IK, and Isolde Sommer) independently rated all items. Items rated 1‐2 by all were excluded, and the highest-rated per subcategory were retained, aiming for about 50 items for the Delphi study.

### Expert Delphi Process

We conducted an expert Delphi study to reach consensus on the most relevant checklist items [21,22]. Experts with backgrounds in patient communication, health information, and online communication were identified through internet searches, existing contacts, team suggestions, and snowballing. Eligible experts were contacted via email and invited to participate. Of the 12 invited German-speaking experts from Germany and Austria, 6 participated (4 from Austria and 2 from Germany; 4 women and 2 men).

In an initial online meeting (May 2022), we presented the study aims, procedures, and categories of the preliminary item list. The experts rated each item’s appropriateness and applicability (1‐5 scale) using an Excel matrix, suggested rewording, and proposed new items. The mean, SD, and range were calculated for each item, and revisions were reviewed in a second Delphi round. The final selection was based on the expert ratings and content relevance. Only minor wording changes were suggested. The result of the 2-step Delphi study was an interim checklist version 1.

### Cognitive Interviews

We used cognitive interviewing to test the interim checklist version 1 with laypersons, focusing on item comprehension and usability. Both think-aloud and verbal probing techniques were applied [23,24].

#### Participant Selection and Sample

Participants were purposively sampled using a maximum variation strategy [25] to reflect diversity in age, gender, education, and migrant background. Eligible were adults (aged ≥18 years) of any gender with a basic education and no formal health-related training, who regularly searched for health information on websites, were fluent in German, and had no cognitive limitations. Recruitment strategies included distributing a study invitation flyer via personal social media channels (Facebook and WhatsApp) and the Medizin-Transparent platforms (Facebook and Twitter), using established contacts with self-help groups, and drawing on our personal networks. In total, 19 interviews were conducted.

#### Data Collection

Interviews were conducted via Microsoft Teams (n=16) or face-to-face (n=3) and were audio-recorded and transcribed. An interview guide and a visually formatted version of the interim checklist version 1 were used. Participants were sent a link to a sample health information web page in advance and were asked during the interview to apply the checklist to this web page, comment on problematic wording, and evaluate the checklist’s layout, usability, and overall quality. Interviewers also completed a structured observation protocol. Interviews lasted 33‐91 minutes (mean 56, SD 14 minutes). Data saturation was reached during the final interviews, with no new relevant issues emerging.

#### Data Analysis

We analyzed the data using the framework approach [26]. In total, 3 researchers (EK, CK-K, and UG) identified and summarized any problems (eg, comprehension, layout, and instructions) in a structured matrix and discussed any uncertainties and revision suggestions in several rounds. Cognitive testing was conducted in 2 waves; after approximately half of the interviews, the checklist items were revised and reordered, and explanatory subtitles were added. The final analysis led to the interim checklist version 2, which served as the basis for the subsequent application testing with additional lay users.

### Application Tests

#### Application Testing With Lay Users

We evaluated the interim checklist version 2 with 20 additional lay users. Participants applied the checklist to 15 purposively selected health information web pages drawn from a larger set of 100 web pages representing both correct and incorrect information on the health intervention. After submitting their checklists, the participants completed a short online questionnaire rating the ease of use (from 1=very easy to 10=very difficult), identifying difficult or important items, and suggesting wording changes. Each participant received approximately US $265 voucher.

The quantitative (descriptive) and qualitative (content-analytic) results informed the final item selection. Fleiss κ was calculated for each item to identify those with a low interrater agreement and limited suitability for lay assessment.

#### Application Testing With Research Team Members

Research team members (BL, IK, DL, BK, IM, and UG) also applied the checklist to all 100 web pages. Two of these researchers independently rated each web page, with disagreements resolved through discussion. The agreement of the dual reviewer ratings per item ranged from 66 to 97 of the 100 web pages. Trustworthiness was defined as answering “yes” to 16‐23 items.

Researchers documented the perceived item difficulty, importance, and ambiguity. These qualitative observations, together with the mean differences between the expert and lay ratings, contributed to the final selection of checklist items.

### Predictive Validity With a Test Set and Creating the Final Checklist

#### Operationalization of Factual Correctness as a Proxy for Trustworthiness

Developing an objective operationalization of trustworthiness is challenging. After extensive discussion within the research team, we concluded that factual correctness was the only feasible proxy for trustworthiness in this context.

Therefore, to assess the predictive validity, we operationalized trustworthiness as the factual correctness of a web page’s answer to a given health question (ie, health claim). We compiled a test set of 100 web pages covering 10 common health questions, with approximately half providing correct and half incorrect information (see Table S8 in Multimedia Appendix 1 for a full list).

The factual strength of evidence for these 10 questions had been established through recent evidence syntheses conducted by Medizin transparent [27], a certified signatory of the International Fact Checking Network at the Poynter Institute [28]. To summarize the evidence behind each health claim, the team uses an ultra-rapid evidence synthesis method [29], using systematic searches, critical appraisal, and Grading of Recommendations, Assessment, Development, and Evaluation ratings [30]. We translated these ratings into a 7-point scale (−3 to 3), with positive values indicating increasing evidence for effectiveness, negative values indicating evidence for ineffectiveness, and 0 indicating insufficient evidence for the health claim.

We developed a parallel 7-point scale to rate the claimed strength of the evidence on each web page, based on commonly used lay terminology. Two researchers independently rated all the web pages, resolving disagreements through discussion.

Factual correctness was then defined as the difference between the factual and claimed strength of evidence. The web pages were classified as incorrect when this difference exceeded 1, while differences of 0 or 1 were considered correct. When the factual evidence was insufficient (0), the web pages implying possible effectiveness or ineffectiveness (±1) were also classified as incorrect.

#### Statistical Analysis for Predictive Validity

We examined which checklist items best predicted the factual correctness of health information on a web page. Using the item ratings from the application tests and the correctness ratings from the test set of 100 web pages, we estimated each item’s predictive validity with a Bayesian logistic regression model (brms in R) [31]. The dependent variable indicated whether a web page provided correct information (1) or incorrect information (0). The model included an interaction between each item and its rating to allow item-specific effects.

#### Creating the Final Checklist

The final item selection was based on each item’s predictive validity, supplemented by the laypersons’ assessments (difficulty, importance, and comments), research team observations, and quantitative indicators of the item performance (interrater agreement and differences between the expert and lay ratings). All factors were considered collectively in determining the final checklist items. This means that no single factor was decisive for inclusion in the final checklist; rather, decisions were based on the combined results, allowing for triangulation across the different inputs.

To support practical application, we created brief explanatory notes for each item and made the checklist with explanations available on the iWISE project website [32]. For this paper, both the items and explanations were translated from German to English: 1 team member drafted the translation, and the full team reviewed, commented on, and finalized it through group discussion.

## Results

### Literature Search and Selection

**Figure 2.**
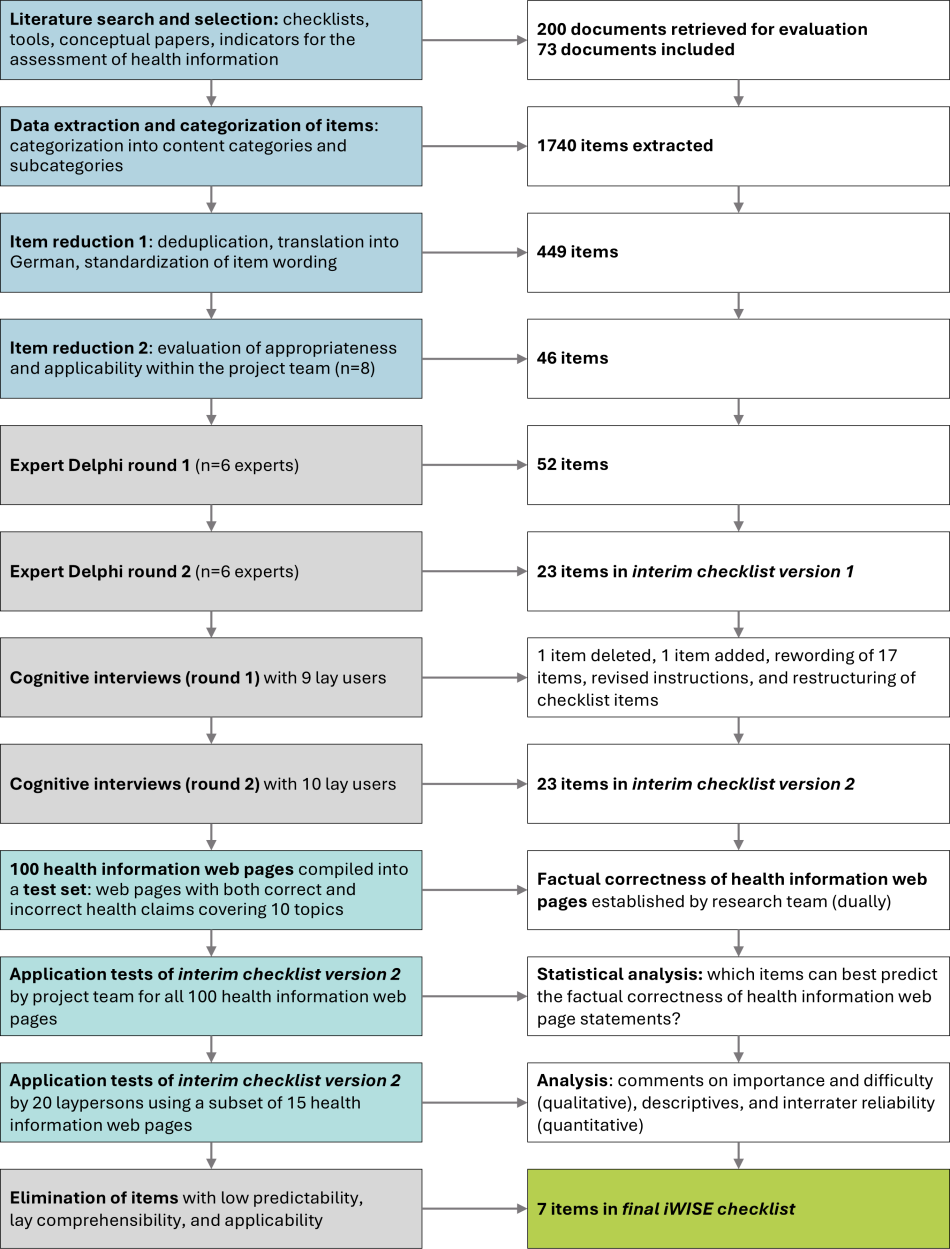
Flow diagram showing the methodological development steps (left) and corresponding sample sizes (right) for the mixed methods study to develop the Info Without Side Effects (iWISE) checklist, involving experts, lay persons, and a formal validation phase.

The database and web searches identified 200 documents that were further evaluated. We also identified 11 relevant reviews or pertinent papers, of which we checked the included tools and checklists (Table S2 in Multimedia Appendix 1). Altogether, 73 documents met our eligibility criteria and were included for data extraction. Figure 2 shows a flowchart of all the documents and items through the development process of the iWISE checklist.

### Data Extraction and Categorization of the Items

We performed a data extraction of the general information for all 73 included documents. Of these, 36 were journal papers, 27 were web pages, 3 were books or book sections, 4 were reports, and 2 were conference papers, and they spanned the literature from 1997 to 2021 (see Tables S3 and S4 in Multimedia Appendix 1for a complete list of all included references). Of the 73 documents, 46 (63%) were tools or checklists, more than half of which were aimed at laypersons, consumers, or patients (27/46, 59%), and approximately one-third at experts (13/46, 28%), that is, health information producers or health care professionals, and 3 (7%) did not have a specific target audience.

We extracted a total of 1740 items and categorized them into 5 content categories or marked them as excluded. Table 1 shows the subcategories for each category along with a description. The largest number of items (450/1740, 26%) belonged to the transparency category, followed by the presentation of information category (402/1740, 23%). The least number of items belonged to the functional or technical aspects category (163/1740, 9%), which is unsurprising, as this was not our search’s main focus (see Table S1 in Multimedia Appendix 1 for the number of items in each subcategory and respective examples). About 14% (237/1740) of the items had to be excluded, either because of the wrong scope, the need for prior knowledge, the lack of generalizability, or the need to use an external source to apply the item, all of which we defined a priori as reasons for exclusion.

**Table 1.** Name and description of the categories and subcategories for the items for evaluating health information on websites extracted from the literature.

Category and subcategory	Description
Functional or technical aspects (deduplicated items: 25; used in the first Delphi round: 0)	
F.0 General technical aspects	A broad category if the item is generally about technology, or several technical aspects are covered at once
F.1 Technical accessibility	Technical accessibility of the website
F.2 Navigation	Navigation through the website
F.3 Interactivity	Interactive offers on the site
F.4 Accessibility or personalization	Possibility of customization (of the presentation) to individual needs
F.5 Other (technology)	An item belongs in this category if it does not fit into any of the available subcategories
Transparency (deduplicated items: 199; used in the first Delphi round: 9)	
T.0 General transparency	A category if the item is generally about transparency and cannot be assigned to any of the subcategories (or must be assigned to several)
*T.1 Up-to-date information*^a^	Information on the content’s actuality is available
*T.2 Author and copyright*	Identification of the content authorship or creator, copyright information
T.3 Author information	Further information that provides an indication of the content creator’s (author’s) context
*T.4 Disclosure and funding*	Identification of funding sources, cooperation partners, conflicts of interest, identification of advertising
*T.5 Standards or certificates*	Information on whether the content creators adhere to certain external guidelines or whether the source is externally certified
T.6 Privacy and data protection	Information or functions regarding privacy and data protection
T.7 Target group	Definition of the website’s target group
T.8 Purpose	Explicit mention of the website’s purpose and goals
T.9 Other (transparency)	An item belongs in this category if it does not fit into any of the available subcategories
Presentation of information (deduplicated items: 66; used in the first Delphi round: 22)	
I.0 General presentation of information	A category if the item is generally about the presentation of information and cannot be assigned to any of the subcategories
*I.1 Balance*	Presentation of information is one-sided, or different aspects are highlighted
*I.2 References*	Details on the origin of the information provided are available
*I.3 Level of evidence*	Information on the categorization of the significance or evidence level or quality of the information is available
*I.4 Quality assurance*	Presence of quality assurance processes
*I.5 Methods*	The procedure for creating the content is described
*I.6 Further information*	Indication of further websites, literature, contacts
I.7 Other (presentation of information)	Not meant are cited sources or the author’s contact information
Presentation (linguistic and visual) (deduplicated items: 99; used in the first Delphi round: 7)	
D.0 General presentation	A category if the item is generally about visual and linguistic presentation or if several subcriteria are covered at once
*D.1 Comprehensibility*	Appropriate presentation of information for the target group (content and form)
*D.2 Layout*	The website and content’s layout and design
*D.3 Linguistic style*	Type of information preparation (neutral, emotional, sensationalist)
D.4 Formal correctness	Reference to the care taken in content creation: are there formal errors in the texts or presentation?
D.5 Other (presentation)	An item belongs in this category if it does not fit into any of the available subcategories
User perception (deduplicated items: 60; used in the first Delphi round: 8)	
N.0 General user perception	A category if the item is generally about user perception and cannot be assigned to any of the subcategories
*N.1 Emotion*	What feelings does the health information trigger?
*N.2 Familiarity and reputation*	Is the source already known? How are the source’s and site creator’s reputation assessed?
*N.3 Trustworthiness of content*	Individual assessment of the content’s trustworthiness
*N.4 Trustworthiness of references*	Individual assessment of the trustworthiness of the sources and of further links
N.5 Trustworthiness in general	General assessment of the trustworthiness of the entire website or the entire offer
N.6 Relevance or usefulness	Individual assessment of the content’s usefulness or practical applicability
N.7 Other (user perception)	An item belongs in this category if it does not fit into any of the available subcategories

a Italicized subcategories contained items used in the first Delphi round.

### Item Reduction

The first step in consolidating the item list resulted in a reduction from 1740 to 449 items. A further reduction step using the research team members’ ratings of the items’ appropriateness and applicability resulted in a list of 46 items (see Table S1 in Multimedia Appendix 1 for the content distribution).

### Expert Delphi Process

The descriptive results of the 46 items rated in Delphi round 1 are shown in Table S5 in Multimedia Appendix 1. Based on the expert feedback, the items were revised from questions to statements, and 6 new items were suggested. In round 2, the experts rated 52 items (the revised 46 plus 6 additions); the results are provided in Table S6 in Multimedia Appendix 1. The applicability ratings showed greater variation than the appropriateness ratings across the rounds (Table S7 in Multimedia Appendix 1).

After completing both rounds, the research team met to discuss the results and to select items. No fixed a priori criteria were set, as the decisions required a comprehensive review of the data. The experts generally rated appropriateness higher than applicability (mean ranges: 3.2‐5.0 vs 2.2‐4.8). We used a stepwise selection approach: first excluding items with a mean appropriateness ≤4.0 or applicability ≤3.0, then removing items with an applicability from 3.0 to 4.0. We ensured that all content categories remained represented and reinstated the highest-rated item where necessary (eg, an item on quality assurance with an applicability rating of 3.17). Redundant items were removed.

This process resulted in 23 items forming the interim checklist version 1. The selected items had higher average ratings for both appropriateness and applicability than the excluded items (Table 2).

**Table 2.** Results of expert Delphi round 2 for the items for evaluating health information on websites; ratings for each item subcategory presented separately for selected and nonselected items.

Category and subcategory	Items not selected			Items selected		
	Items, n	Appropriateness, mean (SD)	Applicability, mean (SD)	Items, n	Appropriateness, mean (SD)	Applicability, mean (SD)
Presentation (linguistic and visual)						
D.1 Comprehensibility	2	3.52 (1.25)	3.27 (0.98)	1	4.00 (1.15)	4.50 (0.76)
D.2 Layout	1	3.67 (0.75)	4.17 (0.69)	0	—^a^	—
D.3 Linguistic style	3	4.17 (0.80)	3.50 (0.73)	1	4.33 (0.75)	4.17 (0.69)
Presentation of information						
I.1. Balance	9	4.35 (0.64)	3.35 (0.98)	7	4.53 (0.73)	4.33 (0.76)
I.2 References	2	4.58 (0.75)	2.83 (0.64)	1	4.67 (0.75)	3.67 (0.94)
I.3 Level of evidence	1	4.67 (0.75)	3.67 (0.75)	1	4.83 (0.37)	3.67 (0.94)
I.4 Quality assurance	0	—	—	1	4.00 (0.82)	3.17 (0.79)
I.5 Methods	1	4.50 (1.12)	3.17 (1.07)	2	4.58 (0.79)	3.67 (0.79)
I.6 Further information	0	—	—	1	4.33 (0.75)	4.67 (0.47)
User perception						
N.1 Emotion	0	—	—	1	4.50 (0.76)	4.17 (0.90)
N.2 Familiarity and reputation	1	4.17 (1.07)	2.83 (0.69)	0	—	—
N.3 Trustworthiness of content	2	4.17 (0.70)	3.25 (0.54)	1	4.00 (0.82)	4.00 (1.15)
N.4 Trustworthiness of references	1	5.00 (0.00)	3.67 (1.11)	0	—	—
N.5 Trustworthiness in general	1	4.17 (0.69)	3.83 (0.69)	1	4.00 (0.82)	4.00 (0.82)
Transparency						
T.1 Up-to-date information	1	4.50 (0.50)	4.33 (0.75)	1	5.00 (0.00)	4.83 (0.37)
T.2 Author and copyright	1	4.67 (0.47)	3.83 (0.69)	2	4.67 (0.47)	4.42 (0.68)
T.4 Disclosure and funding	3	4.56 (0.67)	3.50 (0.97)	1	4.83 (0.37)	4.17 (0.90)
T.5 Standards or certificates	0	—	—	1	4.33 (0.94)	4.33 (0.75)

a Not applicable.

### Cognitive Interviews

We conducted 2 rounds of cognitive interviews, involving 9 persons in the first round and 10 in the second. We managed to recruit a diverse set of persons for inclusion, with 58% (11/19) women, 58% (11/19) between 40 and 64 years of age, 11% (2/19) and 26% (5/19) with a lower educational background of either compulsory school only or apprenticeship or vocational secondary school, respectively, and 21% (4/19) with a migration background (Table 3).

The first-round results showed that many words used in the interim checklist version 1 were difficult to understand for the users, and some items were rated as less important to the lay users than to the experts. Therefore, we made large modifications to the checklist: we reworded 17 of the 23 items, deleted 1 item, and added another; we revised the instructions on how to interpret the checklist results; and we reordered the items in a way in which the information on a web page would usually be presented to avoid too much scrolling by the users.

In the second round, further modifications to the interim checklist version 1 were implemented, for example, adding subtitles to structure the checklist and provide more guidance to the users. Two additional items were tested with the last 7 cognitive interviews.

After analysis and discussions among the whole research team, a second interim checklist version was created for use in the application test (Table 4). The wording of 13 items was changed, or text was added to the item to give tips on where to find the respective information on the web page. Furthermore, the order of the items was changed once more.

**Table 3.** Characteristics of the participants in cognitive interviews assessing the comprehensibility and applicability of interim checklist version 1, and in application tests with lay users evaluating the applicability of interim checklist version 2 of the iWISE^a^ checklist.

	Cognitive interviews (n=19), n (%)	Application tests (n=20), n (%)
Gender		
Women	11 (58)	12 (60)
Men	8 (42)	8 (40)
Age groups (years)		
19‐39	5^b^ (26)	9 (45)
40‐64	11 (58)	10 (50)
65‐84	3 (16)	1 (5)
Highest education level		
Compulsory school	2 (11)	1 (5)
Apprenticeship or vocational secondary school	5 (26)	10 (50)
Upper secondary school or vocational upper secondary school	8 (42)	8 (40)
College or academy or university	4 (21)	1 (5)
Migration background		
No	15 (79)	20 (100)
Yes	4^c^ (21)	0 (0)

a iWISE: Info Without Side Effects.

b One participant was aged 16 years.

c Germany, Turkey, Hungary, and the United States.

**Table 4.** Literal item wording for interim checklist version 2 items for evaluating health information on websites, with the results of the application test with lay users and interrater agreement measures.

	Item text	Items perceived as difficult to answer by lay users (n=20), n (%)	Items perceived as important by lay users (n=20), n (%)	Regression coefficient	Error	96% CI^a^	Agreement among lay users (Fleiss κ)	Agreement among research team members^b^	Item part of the final checklist with wording
1	The title or subtitle is factual and neutral.	1 (5)	0 (0)	0.56	0.24	0.07 to 1.02	0.30	80	^c^-
2	The health information does not contain advertising related to the health problem.	1 (5)	4 (20)	0.46	0.25	−0.02 to 0.94	0.52	85	-
3	The health information does not contain any advertising.	3 (15)	4 (20)	0.82	0.31	0.22 to 1.44	0.50	84	The health information does not contain advertising.
4	The health information is from an independent institution that is unlikely to make money from others’ health. For example, not from a company that sells medicines or food supplements. Tip**:** Such information is often found in the imprint.	6 (30)	14 (70)^d^	0.67	0.24	0.2 to 1.14	0.48	81	The health information is from an independent institution that presumably does not make any money from our health (eg, no suppliers of medicines or food supplements).
5	There is a quality seal on the website. Examples of reliable quality seals are: the Action Forum Health Information System (AFGIS) logo and the International Fact Checking Network.	5 (25)	1 (5)	1.16	0.45	0.27 to 2.06	0.39	94	-
6	I feel the information is presented in a balanced way. For example, different treatment options are described. Or besides positive effects, side effects or disadvantages are also described.	1 (5)	11 (55)^d^	1.12	0.28	0.57 to 1.66	0.30	72	I feel the information is presented in a balanced way (the health information describes, eg, advantages and disadvantages and different treatment options).
7	The health information describes treatment effects that I can feel myself. For example, “the feeling of dizziness decreases” instead of “blood pressure decreases.”	6 (30)	1 (5)	0.48	0.23	0.04 to 0.93	0.16	66	-
8	The health information also tells me if and what consequences there are if I choose not to have a treatment.	8 (40)^e^	1 (5)	0.52	0.47	−0.42 to 1.4	0.08	74	-
9	The health information mentions whether there are differences between men and women. For example, differences in a treatment’s symptoms, effects, or side effects.	4 (20)	2 (10)	0.35	0.45	−0.53 to 1.21	0.22	87	-
10	The health information does not make exaggerated or sensational statements.	1 (5)	1 (5)	0.5	0.25	0.01 to 0.97	0.28	72	-
11	The language is factual and neutral.	0 (0)	5 (25)	0.43	0.24	−0.05 to 0.9	0.12	72	-
12	Technical terms are used sparingly, and their meanings are explained.	0 (0)	6 (30)	0.67	0.26	0.16 to 1.2	0.23	78	Technical terms are used sparingly, and their meaning is explained.
13	It is clear to which target group the health information applies. For example, for men or women, people with certain conditions, etc.	3 (15)	1 (5)	0.24	0.23	−0.23 to 0.68	0.09	76	-
14	The health information indicates how well a fact is scientifically proven.	7 (35)	11 (55)^d^	0.87	0.24	0.4 to 1.34	0.30	80	The health information indicates how well the facts claimed are scientifically supported.
15	The health information provides detailed references for the facts mentioned. For example, a list of sources or links to the studies mentioned.	2 (10)	7 (35)	0.69	0.27	0.16 to 1.2	0.62	85	The health information states in detail which sources are behind the facts mentioned (bibliography, links to studies, etc).
16	The health information clearly states that only a doctor can assess my health problem.	0 (0)	4 (20)	0.7	0.3	0.12 to 1.29	0.41	75	-
17	After reading the health information, I feel I can make a decision without pressure.	4 (20)	1 (5)	0.53	0.24	0.06 to 1.01	0.10	67	-
18	It is clear when the health information was created or updated.	3 (15)	4 (20)	0.68	0.24	0.21 to 1.16	0.67	94	It is clear when the health information was created or updated.
19	It is clear that the health information is up to date. For example, the date it was created or updated is provided.	4 (20)	4 (20)	0.7	0.26	0.19 to 1.19	0.39	88	-
20	It is clear that the health information was written by a person or team with suitable scientific training. For example, medical studies or other health-related training (nursing, pharmacy, biology, etc). Tip: Such information is often found on the “About Us” page or in further links.	5 (25)	9 (45)^d^	0.62	0.34	−0.06 to 1.28	0.34	86	-
21	It is clear that the health information has been checked by a person with appropriate scientific training. For example, medical studies or other health-related training (nursing, pharmacy, biology, etc). Tip: Such information is often found on the “About Us” page or in further links.	9 (45)^e^	6 (30)	0.43	0.45	−0.48 to 1.3	0.31	86	-
22	It is described how the information was created. For example, which studies were considered and why, and which were not. Tip: Such information is often found on the “About Us” page or in further links.	8 (40*)*^e^	1 (5)	1.49	0.52	0.49 to 2.53	0.22	85	-
23	The health information states whether how readers were involved in creating the information. Tip: Such information is often found on the “About Us” page or in further links.	11 (55*)*^e^	0 (0)	3.39	1.07	1.57 to 5.7^f^	0.02	97	-

a Higher values denote better predictive validity.

b Before the disagreements were solved through discussion or by involving a third person; 100=complete agreement.

c Not applicable.

d Items were perceived as important by 45% or more of the lay users.

e Items were perceived as difficult by 40% or more of the lay users.

f This item was only ticked in 8 health information web pages; therefore, the practical value of the predictive validity is low.

Already in the first round, lay users made remarks on single items that were less important to them than to the experts, for example, “It is clear to which target group the health information applies.” As this was an item deemed important by the experts in the Delphi study, we kept it for the application test but added an example because the term target group was ambiguous for some users. In general, many of the suggested modifications led to shorter and more straightforward sentences and the explicit use of examples (ie, “for example”). We also removed redundancies and used words that were unlikely to be misunderstood, and we avoided negative constructions, as these are difficult to understand in German. During the analysis, we realized that some items may be more important for health information creators than for health information consumers, for example, one item that we dropped after the cognitive interviews: “The health information mentions how well the differences between men and women have been researched.”

### Application Tests With Lay Users

We conducted application tests with 20 further lay users (see participant characteristics in Table 3). All 20 lay users returned quantitative and qualitative feedback on using the interim checklist version 2. The mean answer to the general question “On a scale from 1 to 10: how easy or difficult did you find it to use the checklist?” (1=very easy and 10=very difficult) was 4.0 (SD 1.8; range 2-9). We asked the participants to check the items they found difficult to answer, and 4 items were marked by either 8, 9, or 11 participants (40%‐55%; Table 4). Explanations as to why these items were difficult were also given. All this information was taken into consideration in our discussion on the final selection of items for the checklist. As evidenced in Table 4, none of the 4 items that were considered difficult by nearly half or more than half of the lay users made it to the final checklist. The reasons for the difficulties in applying certain items were manifold. For example, regarding item 23 “The health information states whether and how readers were involved in creating the information,” over half of the lay users stated that it was either difficult to find that information on the web page, or the concept of reader involvement in creating health information was unclear to them. Furthermore, no one rated this item as especially important.

Conversely, 4 items were also rated as especially important by 9, 11, or 14 participants (45%‐70%; Table 4), and 3 of these made it to the final checklist. Multiple users had trouble understanding item 14 “The health information indicates how well a fact is scientifically proven,” and 7 also marked it as a difficult item. However, more than half also rated it as especially important, and this item made it to the final checklist.

For item 19 “It is clear that the health information is up to date,” one-third of the participants demanded a clear time frame for their judgment of up-to-dateness. Since the time frame depends on the health information topic and is highly variable, this item was not included in the final checklist, but a more neutral item on currency was included (“It is clear when the health information was created or updated.”).

### Predictive Validity With a Test Set and Creating the Final Checklist

Of the 100 health information web pages in the test set, 37 were factually correct; that is, had little or no difference between the claimed and factual strength of evidence; 63 web pages were incorrect. The regression coefficients of the model for predicting the correctness of the health information web pages are shown in Table 4, along with the estimated errors and 96% CIs. The higher the values, the better the respective item’s predictive validity in predicting the trustworthiness of a health information web page.

Table 4 also shows the agreement among the lay users and among the experts, respectively. Based on the initial results, we tested a few item combinations until we arrived at the final checklist (Textbox 1). As stated earlier, all the quantitative and qualitative results were integrated into our final decision.

Textbox 1. **Seven items of the final Info Without Side Effects (iWISE) checklist.**

1. The health information does not contain advertising.
2. I feel the information is presented in a balanced way (the health information describes, for example, advantages and disadvantages, different treatment options, ...).
3. Technical terms are used sparingly, and their meaning is explained.
4. The health information is from an independent institution that presumably does not make any money from our health (e.g., no suppliers of medicines or food supplements, ...).
5. The health information states in detail which sources are behind the facts mentioned (bibliography, links to studies, ...).
6. The health information indicates how well the facts claimed are scientifically supported.
7. It is clear when the health information was created or updated.

Some items with the highest regression coefficients for predicting a correct health information web page were not chosen because of other reasons. For example, item 23 “The health information states whether and how readers were involved in creating the information” with the highest predictability values was only fulfilled in 8 of 100 health information web pages. Therefore, the practical value of this item’s predictive validity is low. Furthermore, more than half of the lay users perceived this item as difficult to answer, and the agreement among the lay users in answering this item was very low (Table 4). The situation was similar with item 22: “It is described how the information was created. For example, which studies were considered and why, and which were not.” While this had a good predictive value, 8 of 20 lay users perceived it to be difficult to answer; therefore, we decided not to include it in the final checklist, as only items that are easily answered and clear to laypeople are useful in the final checklist.

After several discussion rounds among the whole research team, we arrived at the final iWISE checklist with 7 items (Textbox 1).

According to our statistical model, the probability that the health information is correct if all of these items are marked with “yes” is 99.9%. The probability that the health information is correct if all of the items are marked with “no” is 20.4%. However, this requires that the items are marked correctly by the user, which, in practice, may not be the case.

We published the iWISE checklist in German on our project website [32] and also provided brief explanatory notes for each item to help users apply the checklist. We also created a video explaining all 7 items. The English version of the iWISE checklist, including explanations for lay users, is available in Multimedia Appendix 1 and can also be downloaded as a PDF [33].

## Discussion

### Main Study Findings

In this mixed methods study, we developed and validated the iWISE checklist, a concise and easy-to-use tool enabling laypersons to evaluate the trustworthiness of health information on websites. To our best knowledge, iWISE is the first checklist that can be applied by laypersons in everyday information-seeking situations without the need for prior training. Consistent with our aims, we developed iWISE in a multistage development process, combining evidence synthesis, expert review, iterative cognitive testing, application tests, and predictive validation, which resulted in 7 items that lay users can both understand and reliably apply. To further support lay users, we also provide brief, plain-language explanations for each item, including examples and tips on where to find the relevant information on a web page.

The final iWISE checklist contains 7 items that assess key aspects of health information trustworthiness, including the absence of advertising, a balanced presentation of information, the limited use of professional jargon, an origination from an independent organization, the citation of sources, the mention of scientific validation, and the presence of a publication date. The checklist demonstrated the ability to distinguish between evidence-based and nonevidence-based health information web pages in the German language: the validation testing showed that when all items were marked with “yes,” there was a nearly 100% probability that the health information was also factually correct.

### Discussion and Comparison With Prior Work

There are 3 main features that characterize the iWISE checklist: it is more user-friendly for laypersons than prior checklists, it was developed with the extensive involvement of laypersons, and it reliably identifies trustworthy health information on web pages, as demonstrated by the validation.

#### iWISE Is Shorter, Simpler, and More Practical Than Prior Tools

The iWISE checklist is short and easy for lay users to understand and apply when judging the trustworthiness of health information on websites. Numerous tools and checklists are available to assess online health information [12,34-36]; however, these tools were either prepared for research purposes or for use by health care professionals, have not been developed by considering the lay user perspective, contain a large number of items, or have sophisticated scoring systems that make them impractical for consumers to use [11,22,37,38]. Others are intended for written consumer information, such as the DISCERN instrument [39,40] or the Ensuring Quality Information for Patients tool [41,42], neither of which can be directly transferred to health information web pages. More broadly, the existing approaches to rating health-related websites [43] range from provider-focused quality criteria that contain a list of recommendations for website development and content [44-48], to quality labels or logos that are displayed on screen and represent a provider’s commitment to implement or adhere to a set of quality criteria such as the Health On the Net code [49] (which was permanently discontinued in December 2022), to user guidance systems requiring users to navigate long lists of questions [11,12,22,37,38]. These approaches often require more time, expertise, or effort than laypersons can invest during everyday health information-seeking. In contrast, iWISE consists of 7 core items that reflect the key aspects of credibility and quality but remain manageable within typical cognitive limits for lay users, consistent with research on short-term memory capacity [50].

#### iWISE Was Developed With the Extensive Involvement of Laypersons and Is Tailored to Their Needs

A major strength of iWISE is that lay users were integrated throughout the development process, using cognitive interviews and application testing, and these results were taken into account in the final item selection. Other quality evaluation checklists are mostly based on expert views [51] and, therefore, may not meet the needs of laypersons with a low general education level and no formal health-related education. For example, the MAPPinfo checklist includes items that are justified by either ethics or research evidence, which enhances the methodological rigor but may omit criteria important to users that lack strong research backing [11]. Furthermore, MAPPinfo was user-tested with nursing and medical students rather than true laypersons. In contrast, iWISE was built explicitly for individuals with no specialized health knowledge, including people with lower educational backgrounds. Items requiring prior expertise were eliminated, ensuring that all the remaining items could be answered solely by examining the web page itself. Because a checklist must be usable in real-world situations—often without printing or switching between windows—the final 7 items were intentionally kept short, intuitive, and memorable. The final content of the checklist is also closely aligned with criteria lay users naturally use, as described by Sun et al [51], including trustworthiness, expertise, and objectivity.

#### iWISE Effectively Identifies Trustworthy Online Health Information

The validation test demonstrated that the 7 iWISE items help distinguish between evidence-based and nonevidence-based web pages on health interventions. The validation method—operationalizing trustworthiness as the factual correctness of the web page’s claims—is novel and unique in this field. The factual correctness was fulfilled if the factual strength of the evidence for a certain health question and the claimed strength of the evidence on the web page were the same or only differed slightly. For our validation, we applied the Grading of Recommendations, Assessment, Development and Evaluation system [30], which is widely used in evidence-based medicine. Only items that showed a reasonable association with factual correctness were retained for the final checklist. Factual correctness is also one of the criteria lay users value most highly when evaluating online health information [51]. Importantly, the predictive validity analysis showed that iWISE meets the key criteria for consumer-oriented rating tools described by Hanif et al [52], including readability, feasibility, and practical assessibility.

### Limitations

Our checklist has some limitations. With 100 web pages, our validation test set of health information articles was relatively small. We tested our checklist with a limited set of only 10 popular health questions—and with 10 articles for each of these questions, respectively. Our test set might therefore be biased and not be representative of the broad mass of health information that a typical user encounters when searching the web. A different, more varied, and larger test set might have resulted in a slightly different combination of checklist items. However, iWISE is comparable with other checklists that have either used a smaller validation test set or that deal with one health topic or both, for example, the MAPPinfo checklist was tested on 57 web pages [11], the Quality Evaluation Scoring Tool on 45 online articles on Alzheimer disease treatment and prevention [37], and Dobbins et al [38] used 120 web resources and another 107 in a second validation round on healthy aging.

The predictive validity results, one of the factors we considered in selecting the final checklist items, are based on the ratings from the application test with the research team. The laypersons’ ratings were not integrated in this validation step. However, the perspectives of the laypersons were considered in other ways, and we specifically ensured that all items were well understood by our lay user group.

Our checklist has only been validated for information on treatments or health decisions. It has not been tested on websites lacking interventional information. The checklist might therefore not be valid for judging the accuracy of information on symptoms for the diagnosis of a disease or on the health risks of noninterventional hazard exposure.

The iWISE checklist was tested with German-speaking laypersons in Austria and validated on a test set of websites in the German language. We cannot rule out that the checklist works differently when tested in other languages. In addition, our checklist is not tested with social media postings or health information videos and cannot be used for evaluating medication leaflets.

### Conclusions

The iWISE checklist offers an accessible and empirically validated way for laypersons to judge the trustworthiness of online health information. By reducing a complex evaluation process to 7 intuitive items, iWISE has the potential to strengthen critical health literacy in everyday decision-making. In a digital environment where misinformation is widespread, such an accessible tool can help individuals navigate health claims with greater confidence while also encouraging health information providers to adopt more transparent and balanced communication practices.

Because iWISE was developed and validated in a German-language context, future research should examine its applicability across other languages and cultural settings. Research teams in other countries may wish to validate or adapt the checklist for their own health information environments. In addition, the checklist was developed for web pages and should be tested for use with social media posts and health information videos, where misinformation is especially prevalent. Building on this need, our team has already initiated a follow-up project to develop and evaluate a complementary tool specifically tailored to assessing the reliability of health information on social media platforms.

## Supplementary material

10.2196/69529 Multimedia Appendix 1 Detailed methods description, supplementary results, and tables on the development, and the final Info Without Side Effects (iWISE) checklist.

## Abbreviations

iWISE: Info Without Side Effects
MAPPinfo: Mapping the Quality of Health Information
SRQR: Standards for Reporting Qualitative Research
