# Supplementary material for: Development of a Validated Lay Checklist (Info Without Side Effects) for Assessing Health Information on Websites: Mixed Methods Study
Source: J Med Internet Res. 2026 May 20;28:e69529. doi: 10.2196/69529 (PMC13189254; doi:10.2196/69529)
Supplement: Multimedia Appendix 1 [file jmir-v28-e69529-s001.docx]

# Detailed methods description

This appendix provides a detailed description of the methods used for the development process of the Info without Side Effects (iWISE) checklist, while the main manuscript includes only a brief version.

## Working definitions

### Health information on websites

We defined *health information on websites* as any health-related content accessible on a webpage via an online search engine (e.g., Google). A website consists of multiple webpages under one domain. In this study, we focused on information about health interventions, defined as treatments claimed or believed to relieve or prevent symptoms or diseases (e.g., vitamin C for the treatment of a cold). Health information from social media platforms or AI-generated summaries was not included.

### Trustworthiness of health information on websites

For the purpose of this study, we defined *trustworthiness of health information on websites* as the degree to which the information on a given webpage can be trusted by laypersons to be reliable and valid. This definition aligns with Viviani and Pasi’s [1] conceptualization of trustworthiness as a key dimension of credibility, focusing specifically on the audience’s perception. As laypersons generally do not have the domain expertise to directly judge whether health information is factually correct, we use trustworthiness as a proxy for the correctness, that is, accuracy of the information.

### Laypersons

Following the Cambridge Dictionary [2], we defined *layperson* as someone who is not an expert in or does not have detailed knowledge of health-related topics, that is, persons without a formal health-related education (i.e., no medical doctors, nurses, or other health care professionals or health researchers, etc.).

## Study design

We employed a sequential, multistage mixed-methods design to develop our checklist, in which the findings from each step iteratively informed focus and data collection of subsequent stages [3]. In short, the development process included a comprehensive literature review including extracting possible checklist items, an expert Delphi study, cognitive interviews with lay users, and application tests with laypersons and the research team using a test set of 100 webpages to determine the suitability of potential checklist items for predicting the factual correctness of health information content (see Figure 1 for an overview of the development process).

We developed a protocol a priori and published and registered it in the Open Science Framework (OSF) retrospectively [4]. During the study, we made a few amendments to the protocol. To ensure comprehensive reporting, we adhered to the Standards for Reporting Qualitative Research (SRQR) [5] and the American Psychological Association Style Journal Article Reporting Standards for mixed-methods research [6].

## Ethical considerations

The study followed the University for Continuing Education Krems’ privacy policy (art. 13 GDPR) and was approved by the university’s Ethics Commission (EK GZ 23/2021-2024). Interview participants received written study information and provided verbal informed consent; they received a €30 voucher as compensation. Transcripts were de-identified by replacing all names and other identifiable details with alphanumeric codes.

## Literature search and selection

In a first step, we carried out a literature review for existing tools and/or checklists for the assessment or evaluation of (online) health information as well as for quality criteria and indicators of trustworthy and reliable health information.

While we did not conduct a systematic review, we aimed to identify a broad variety of tools available. Exploratory searches revealed that many relevant tools were grey literature, so we adopted a search process inspired by the Tailored Approach by Cooper et al. [7], which combined a variety of search methods:

- An information specialist (IK) conducted precision-focused searches for reviews about the evaluation of health information in PubMed, Scopus.com, Epistemonikos.org, and Library, Information Science & Technology Abstracts (searched via Ebsco).
- Several team members (IK, BK, FKA, CK, BL, EK, UG) carried out independent web searches using Google, Google Scholar, and the Bielefeld Academic Search Engine (BASE).
- The reference lists of all the identified (systematic) reviews were checked for citations of eligible tools or publications.

All searches were conducted between March and September 2021. The search results were collected in an Endnote 20 Library and checked for duplicates.

One person screened all the results, and a second person checked the selected documents for completeness.

We included published and grey literature documents in the English or German language (i.e., journal articles, reports, webpages, preprints) from the following categories:

- Checklists and tools for the evaluation of online health and other online information aimed at laypersons or professionals
- Research on the concepts, quality criteria, and indicators relevant for the evaluation of online health information
- Systematic reviews of the tools/checklists or concepts/quality criteria/indicators

From each included systematic or literature review on tools and checklists, we extracted the tools’ and checklists’ names and references, and we included the references that were included in more than three reviews.

Because of the varied nature of the included documents (e.g., published articles, webpages, downloadable PDFs of checklists, tools, and grey literature reports, etc.), we could not adhere to a strict separation of abstract and full-text screening. Each document was screened by one person, with a second person verifying the inclusion/exclusion decision.

We excluded literature that either exclusively described the evaluation of the layout, design, comprehensibility, or readability of (online) health information as well as literature applicable only for evaluating health information for specific diseases and point-of-care information.

## Data extraction

We developed a data extraction matrix in Microsoft Excel to facilitate the data extraction and item generation step. The data extraction consisted of three parts: 1) general information from the included references, that is, the reference details, reference type, checklist/tool name, target group(s), application field, and validation status; 2) the names of domains and subdomains/subcategories, the literal item text and original answer categories (if available), or the description of the domain/criterion/indicator (if applicable); 3) for each included (systematic) review on tools/checklists, we extracted the included names and references of the tool/checklist and added the most frequently mentioned tools/checklists that we had not included yet.

After the first few data extractions, we discussed uncertainties within the project team and revised the data extraction form accordingly. If references had to be excluded during the data extraction, these decisions were always discussed with another research team member.

## Categorization of the items

The next step after the data extraction was the categorization of the items into content categories. One research team member developed a draft categorization scheme inductively, which we discussed as a team. We developed a categorization scheme iteratively including the names of the overall categories and subcategories, a description of each subcategory, and coding examples (see Table S1).

We excluded all criteria that were unsuitable because they did not comply with the a priori–defined feasibility criteria adapted by Provost 2006 [8] and marked them as “excludes:”

- Timeliness: Criterion must be assessable in a short time.
- Expertise independent: Criterion must be assessable without prior topic-specific knowledge.
- Externability: Criterion must be assessable without using secondary sources.
- Generalizability: Criterion must be applicable to all possible (online) health information (i.e., not focused on specific aspects of a disease or a specific health topic).

We added further subcategories for the “excludes” domain during the process (see Table S1):

- Scope: Content of the item is not within our scope.
- Overall category: Only the item category is specified, no items.
- Unclear: Item is too unclear to be used in a checklist.
- Open question: It is not possible to formulate a yes/no question.

We piloted the categorization process and revised the category system. For each category, there was one subcategory called “other,” where we added items provisionally and either created new subcategories or decided on a subcategory after joint discussion.

Four members of the research team (CK, EK, IK, UG) categorized all the available items, and a second person checked the categorization. Any uncertainties were discussed together.

Table S1: Item categorization by category

| **Subcategory** | **Description** | **Example** | **Number of items after categori-zation (n=1740)** | **Number of items after dedupli-cation & merging (n=449)** | **Number of items for the 1^st^ Delphi round (n=46)** |
| --- | --- | --- | --- | --- | --- |
| **Functional/ technical aspects** |  |  | **163 (9%)** | **25 (6%)** | **NA**^a^ |
| F.0 General technical aspects | A broad category if the item is generally about technology or several technical aspects are covered at once |  | 9 | 0 | NA |
| F.1 Technical accessibility | Technical accessibility of the website | Browser compatibility, website loading times, registration | 40 | 10 | NA |
| F.2 Navigation | Navigation through the website | Site menu, simple navigation, internal search engine | 55 | 5 | NA |
| F.3 Interactivity | Interactive offers on the site | Direct feedback options on the site (e.g., comment field, rating options, chat room, forum, etc.), download options | 38 | 3 | NA |
| F.4 Accessibility/ personalization | Possibility of customization (of the presentation) to individual needs | Customization of font sizes/types, text-only option, several languages available | 17 | 3 | NA |
| F.5 Other (technology) | An item belongs in this category if it does not fit into any of the available subcategories |  | 4 | 1 | NA |
| **Transparency** |  |  | **450 (26%)** | **199 (44%)** | **9 (20%)** |
| T.0 General transparency | A category if the item is generally about transparency and cannot be assigned to any of the subcategories (or must be assigned to several) |  | 82 | 0 | 0 |
| T.1 Up-to-date information | Information on the content’s actuality is available | Date of creation and/or last update stated; update frequency stated | 82 | 31 | 2 |
| T.2 Author and copyright | Identification of the content authorship/ creator, copyright information | Name of the author/authorship, email address, imprint | 81 | 43 | 2 |
| T.3 Author information | Further information that provides an indication of the content creator’s (author’s) context | Mention of the content creator’s role, profession, education, name, author qualifications; mention of affiliation, (type of) institution | 52 | 29 | 0 |
| T.4 Disclosure and funding | Identification of funding sources, cooperation partners, conflicts of interest, identification of advertising | Naming of sponsors, financing institution, adverts/advertising is shown; separation of editorial and advertising content | 126 | 47 | 4 |
| T.5 Standards/certificates | Information on whether the content creators adhere to certain external guidelines or whether the source is externally certified | HONcode Standard | 6 | 4 | 1 |
| T.6 Privacy and data protection | Information/functions regarding privacy and data protection | General disclaimer, information on the use of cookies, message when leaving a secure website | 46 | 24 | 0 |
| T.7 Target group | Definition of the website’s target group | Explicit explanation of who the website and its content are aimed at | 17 | 5 | 0 |
| T.8 Purpose | Explicit mention of the website’s purpose and goals | Educational purposes, commercial interests (this does not refer to subjective assumptions as to what the purpose might be) | 40 | 16 | 0 |
| T.9 Other (transparency) | An item belongs in this category if it does not fit into any of the available subcategories |  | 0 | 0 | 0 |
| **Presentation of information** |  |  | **402 (23%)** | **66 (15%)** | **22 (48%)** |
| I.0 General presentation of information | A category if the item is generally about the presentation of information and cannot be assigned to any of the subcategories |  | 7 | 0 | 0 |
| I.1 Balance | Presentation of information is one-sided, or different aspects are highlighted | Complete, scientifically balanced comparison of information; in addition to benefits, risks, possible complications, and the consequences of no treatment are mentioned, and other treatment options are listed; information and recommendations are clearly distinct | 215 | 34 | 12 |
| I.2 References | Details on the origin of the information provided are available | Availability of information sources, references, links to sources | 66 | 9 | 3 |
| I.3 Level of evidence | Information on the categorization of the significance/evidence level/quality of the information is available | The text explicitly addresses how well or poorly the findings/data are scientifically validated. Gaps in knowledge are addressed | 30 | 10 | 2 |
| I.4 Quality assurance | Presence of quality assurance processes | It is clear to users that there is an editorial review process and how it works | 21 | 4 | 1 |
| I.5 Methods | The procedure for creating the content is described | Transparent systematic literature search, evaluation according to the GRADE criteria | 22 | 4 | 3 |
| I.6 Further information | Indication of further websites, literature, contacts | Links to further content; references to offers of help; disclaimer: note that health information does not replace a doctor visit | 41 | 5 | 1 |
| I.7 Other (presentation of information) | (Not meant are cited sources or the author’s contact information) |  | 0 | 0 | 0 |
| **Presentation (linguistic and visual)** |  |  | **303 (17%)** | **99 (22%)** | **7 (15%)** |
| D.0 General presentation | A category if the item is generally about visual and linguistic presentation or if several subcriteria are covered at once |  | 9 | 2 | 0 |
| D.1 Comprehensibility | Appropriate presentation of information for the target group (content and form) | Appropriateness of language, sentence structure, and readability; comprehensible presentation of figures; explanation of technical terms; degree of complexity is adapted to the target group | 116 | 42 | 1 |
| D.2 Layout | The website and content’s layout and design | The content’s layout (e.g., font, font size, structure) and visual presentation (e.g., illustrations, graphics, tables), clarity (e.g., short text), appropriate color contrast, appealing design, illustration quality, graphics, tables (readability), etc. | 126 | 40 | 1 |
| D.3 Linguistic style | Type of information preparation (neutral, emotional, sensationalist) | Objective, neutral language style; no judgmental, emotionalizing language | 42 | 14 | 5 |
| D.4 Formal correctness | Reference to the care taken in content creation: Are there formal errors in the texts or presentation? | Correct grammar and spelling, figures in text and tables match | 10 | 1 | 0 |
| D.5 Other (presentation) | An item belongs in this category but does not fit into any of the available subcategories |  | 0 | 0 | 0 |
| **User perception** |  |  | **185 (11%)** | **60 (13%)** | **8 (17%)** |
| N.0 General user perception | A category if the item is generally about user perception and cannot be assigned to any of the subcategories |  | 10 | 2 | 0 |
| N.1 Emotion | What feelings does the health information trigger? | Subjective assessment based on the formal and content-related presentation of the information | 7 | 4 | 1 |
| N.2 Familiarity and reputation | Is the source already known? How are the source’s and site creator’s reputation assessed? | Subjective assessment based on the content but also on external factors (prior knowledge, bias, background, etc.) | 40 | 13 | 1 |
| N.3 Trustworthiness of content | Individual assessment of the content’s trustworthiness | Subjective assessment based on the content but also on external factors (prior knowledge, bias, background, etc.) | 47 | 15 | 3 |
| N.4 Trustworthiness of references | Individual assessment of the trustworthiness of the sources and of further links | Subjective assessment of the trustworthiness of specified sources of information and data, of further literature, of references to offers of help | 10 | 5 | 1 |
| N.5 Trustworthiness in general | General assessment of the trustworthiness of the entire website/the entire offer | Trust in the organization creating the offer; assessment of the website name or URL | 29 | 13 | 2 |
| N.6 Relevance/usefulness | Individual assessment of the content’s usefulness/practical applicability | Influence of the information on individual user behavior; relevance to the user’s original need for information; specific examples for users are given | 42 | 8 | 0 |
| N.7 Other (user perception) | An item belongs in this category if it does not fit into any of the available subcategories |  | 0 | 0 | 0 |
| **Excludes** |  |  | **237 (14%)** | **NA**^b^ | **NA**^b^ |
| X_external source | Can only be assessed by using additional tools/sources | If the institution’s or author’s reputation is unknown and further internet research would be necessary | 36 | NA | NA |
| X_Scope | The item’s content is not within this study’s scope | Items on laypersons’ search behavior; items for health information producers | 82 | NA | NA |
| X_Generalizability | The item cannot be generalized, but can only be used for a specific type of health information | The item is only relevant for cancer information; the item refers to a comparison of treatment methods and may not be used for health information on the prevalence of diseases | 59 | NA | NA |

^a^ items from the functional/technical aspects category were excluded as they were deemed not useful for the checklist

^b^ excludes were not further deduplicated or translated

Abbreviations: GRADE, Grading of Recommendations, Assessment, Development and Evaluation; n, number; NA, not applicable

## Item reduction

The first step of condensing the item list was the merging of duplicate and similarly worded items as well as the translation of English items into German. We translated the materials from English to German either directly based on our own linguistic expertise or with the support of online translation tools, and we carefully reviewed all translations for accuracy. As the literal items were very diverse, we also unified their wording in two ways: first, we changed the wording so that each item was a question and, second, we determined that the answer “yes” always indicates a trustworthy text. This was done in one step by one person. We divided all the items among four team researchers (CK, EK, IK, UG) and developed a procedure for merging and item formulation to ensure that the process was done similarly by each. We noted discussion points and discussed ambiguities and uncertainties during the condensation process among the team. After the first round of merging and item formulation, the process was checked by a second person, and discrepancies or conflicts were resolved by discussion.

Our target for the first Delphi round was approximately 50 items, but after merging the duplicates, we still had too many items to conduct a meaningful expert Delphi study. We therefore applied a further reduction step. For this, we created an Excel file matrix with the item text, the category and subcategory allocation, and two rating options on a scale from 1 to 5, with 1 being “very poor appropriateness/applicability” and 5 being “very good appropriateness/ applicability:” one for the item’s appropriateness (i.e., how appropriate is the item for assessing whether the health information is trustworthy) and one for the item’s applicability (i.e., how easily can the item be applied by checklist users without prior knowledge). After being given detailed instructions, eight members of the research group (EK, FKA, BL, UG, BK, CK, IK, IS) determined the ratings separately. We calculated each item’s mean rating to decide which to keep for the expert Delphi study. Items that scored very low (i.e., achieving only ratings of 1 and 2 from all eight research group members) were eliminated without further inspection. All other items within each subcategory were checked to determine which was the highest rated alternative; the other items were eliminated. The final selection of 46 items was made according to content-related aspects and while accounting for the target number of approximately 50 items for the expert Delphi study.

## Expert Delphi process

Next, we conducted an expert Delphi study to solicit the opinions of invited experts and to establish consensus on the most pertinent items for the checklist [9; 10]. We aimed to assemble a diverse panel with expertise in patient communication, health information, and online communication. To identify potential experts, we searched the internet, utilized established contacts, took suggestions from within the research team, and applied a snowballing approach. Experts were contacted via email and invited to participate if they met the inclusion criteria of having academic or professional expertise in patient communication, health information, or online communication. Out of the twelve invited German-speaking experts from Austria and Germany, six participated in the expert Delphi study (four from Austria, two from Germany; four women, two men).

During an initial online meeting with the invited experts in May 2022, we clarified the research objectives and study process and presented the categories and subcategories of the preliminary item checklist. The experts received a comprehensive information sheet as well as instructions for participating in the Delphi study, using the same Excel file matrix layout as in the previous item reduction step. In addition to rating the items from 1 to 5 for appropriateness and applicability, the experts were given the opportunity to suggest improvements in the wording of existing items or to provide further comments. They were also encouraged to propose up to five new items that they felt were missing. We calculated the mean, standard deviation, and minimum and maximum rating values for each item and incorporated suggestions for improvements and ideas for missing items. In the second Delphi round, the expert panel rated the items again for appropriateness and applicability. Descriptive statistics were calculated again, and items were selected based on the experts’ scores and content considerations. The result of the two-step Delphi study was interim checklist version 1.

## Cognitive interviews

Next, the first version of the interim checklist was presented to laypersons in a cognitive interview. The cognitive interview methodology examines individuals’ cognitive processes when processing information and responding to questions [11] and is widely used in questionnaire pretesting [12; 13]. Cognitive interviewing was used in this study to identify problems with item comprehension and to evaluate the usability and applicability of interim checklist version 1. We used both the think-aloud and verbal probing techniques [14; 15], where participants were explicitly instructed to verbalize their thought processes during the cognitive interview.

### Participant selection and sample

The sample size and characteristics were based on the recommendations of the cognitive interview [16]. We used the purposive sampling method and maximum variance strategy for the participant recruitment and selection [17]. Recruitment strategies included distributing a study invitation flyer via personal social media channels (Facebook, WhatsApp) and the *Medizin-Transparent* platforms (Facebook, Twitter), using established contacts with self-help groups and drawing on our personal networks. Interested individuals were asked to provide basic sociodemographic information to populate our sampling grid. We selected participants to reflect the current sociodemographic pattern in terms of age groups, gender, educational level, and migrant background. Eligible participants were adults of any gender, 18 years and older, with a basic education level (i.e., compulsory schooling or high school diploma) and no formal health-related education (i.e., no medical doctors, nurses, or other health care professionals or health researchers, etc.), who regularly use the internet to seek answers to health-related questions (i.e., at least once in the last three months). Because the cognitive interviews were conducted in German, our target group consisted of individuals who are fluent in German and have no cognitive limitations. Before the interview, the participants received an information sheet explaining the purpose of the study, the data privacy policy, and the cognitive interview procedure.

### Data collection and ethical considerations

The interviews were conducted either online via MS Teams (n=16) or face-to-face (n=3), according to the study participants’ preferences. Participants provided their informed consent verbally prior to starting the interviews and received a gift voucher of 30 Euro afterward to compensate for their time and effort.

We developed an interview guide with probing questions prior to the first interview and adapted it after discussion among the research team (see section “Interview guide used in the cognitive interviews”). Furthermore, we prepared a version of interim checklist version 1 with a visually appealing layout along with brief written instructions for use. The interviews were conducted by three researchers (EK, CK, UG), recorded using MS Teams or a recording device, and transcribed verbally. Transcripts were de-identified by replacing all names and other identifiable details with alphanumeric codes. The interviewers preselected three sample health information webpages for the cognitive test. A few days before the interview, the study participants received an email from the interviewer with a link to the respective health information webpage and an invitation to read the text in advance. While responding to the checklist items, participants were free to view the sample health information webpage and to view other pages within the same website. Participants were asked to apply the checklist to the provided health information webpage. They were prompted to comment on any potentially problematic words or phrases in the checklist and whether they had any problems answering the checklist items. Additionally, probing questions were used to identify or explore potential sources of response errors. Finally, the study participants were asked to evaluate the checklist’s layout, usability, and overall quality. The interviews lasted between 33 and 91 minutes (mean: 56 minutes). Further, each interviewer completed a structured protocol on their observations of any problems encountered along with comments on the nature of the problems, or whether there was no evidence of a problem. Data saturation was reached during the final interviews, with no new relevant issues emerging.

### Data analysis

We employed the framework approach [18] to analyze the interview data. The three interviewing researchers (UG, CK, EK) started by reading the transcripts of the interviews they conducted and their notes on the observation sheet. They marked potential problems (e.g., problems with comprehension, knowledge, or the instructions or checklist layout) and extracted the data into a structured matrix. Any reasons for uncertainties, suggestions for revision, or comments on which words and phrases were particularly well understood were extracted into the structured data analysis table. The responses to the additional questions were also paraphrased in the matrix. Each interviewer extracted the data from their own interviews, and the overall assessment was done in several discussion rounds among all three analyzers (UG, CK, EK). Questionable or controversial items were identified and suggestions for any revisions discussed among the whole research team. The cognitive testing was done in two rounds: after approximately half of the interviews, the checklist was adapted, the order of the checklist items was rearranged, and explanatory item subtitles were added. The analysis of all the cognitive interviews and discussions among the whole research team resulted in interim checklist version 2, which was used as the basis for the application testing with additional lay users.

## Application testing with lay users

We tested the applicability of interim checklist version 2 with another set of potential future lay users.

We recruited 20 additional laypersons, including interested individuals not previously selected for the cognitive interviews. Participants were asked to apply the checklist to 15 selected webpages with information about health interventions. After returning the completed checklist documents, they received a link for a short online post-task questionnaire. The 15 health information webpages were a purposively selected sample from a test set consisting of 100 health information webpages (see next section) representing a range of different types of health interventions—around half of which provided the information and underlying evidence correctly and half of which did not. The post-task questionnaire asked participants to rate how easy or difficult they found using the checklist on a scale from 1 (very easy) to 10 (very difficult). It also provided a list of all 23 items from interim checklist version 2, where participants could mark those that were difficult to answer and provide an explanation for why. Furthermore, we asked participants to mark the items that they perceived as particularly important for evaluating health information. Participants were also given the opportunity to suggest the rephrasing of item texts. The interim checklist version 2 items used in the application test can be found in Table 4 in the main manuscript.

Each participant was remunerated for their effort with a voucher of 225 Euro after having returned the completed checklists and questionnaire.

We considered the quantitative and qualitative results from the application testing with lay users in the final selection of items for the final checklist. We calculated Fleiss’ Kappa as an interrater reliability measure for the laypersons’ ratings for each item to see whether they arrived at very different answers. The aim was to identify such items because they are obviously unsuitable for an objective assessment of health information on websites by lay users. The data from the post-task questionnaires was analyzed in two ways: the quantitative data was analyzed descriptively, and the qualitative data in the open answers by means of content analysis.

## Application testing with research team members

Interim checklist version 2 was also applied to the full test set of 100 health information webpages by members of the research team (BL, IK, DL, BK, IM, UG). The health information was considered trustworthy if between 16 and 23 out of a total of 23 items were answered with “yes.” To maximize the objectivity, each webpage was assessed independently by two researchers, and disagreements were resolved by discussion or consultation with a third person. For each item, the agreement of the dual reviewer ratings (before reaching a consented rating) ranged from 66 to 97 (out of a possible 100 webpages from the test set). We used the quantitative data from the checklists (i.e., the item ratings and interrater agreement) for the statistical analysis (see section “Statistical analysis for predictive validity”).

In addition, after the application test, each research team member took notes on the specific item’s difficulty, importance, or possible ambiguity. We also calculated the mean difference between the consented expert ratings and each layperson’s rating.

## Predictive validity with a test set and creating the final checklist

### Operationalization of the trustworthiness of health information on websites

To evaluate the predictive validity, we required a measurable indicator of trustworthiness. We therefore operationalized trustworthiness as the factual correctness of a webpage’s answer to the respective health question. To test the predictive power of our items, we compiled a test set of 100 webpages that provided answers to ten common health questions (e.g., “Does vitamin C help with a cold?”, “Does arthroscopy help with osteoarthritis?”, “Do omega-3 fatty acids prevent cardiovascular diseases?”; see Table S8 for a full list). For each question, we identified ten webpages through a Google search and aimed for a balanced sample in which roughly half of the webpages reflected the evidence base correctly and half did not.

### Assessing the factual and claimed strength of evidence

The underlying strength of evidence for the answers to these ten health questions (i.e., health claims) was systematically researched within the previous year by specially trained science journalists from *Medizin-Transparent* [19], a certified signatory of the International Fact Checking Network at the Poynter Institute [20]. *Medizin transparent* is a project of Cochrane Austria, based at the Department for Evidence-based Medicine and Evaluation at the University for Continuing Education Krems. To summarize the evidence behind each health question, the team uses an ultra-rapid evidence synthesis method [21], starting with a systematic literature search followed by a critical appraisal of the studies’ quality (risk of bias). The strength of the evidence—that is, the reliability of the study findings—is then assessed using the Grading of Recommendations, Assessment, Development and Evaluation (GRADE) approach [22]. GRADE uses four categories to reflect the strength of evidence: very low, low, moderate, and high. When the strength of evidence is “very low,” it remains uncertain whether the intervention in question is effective or ineffective. To reflect these varying degrees of evidence, we developed a 7-point integer scale ranging from ‑3 to 3. Insufficient evidence is coded as 0; evidence for effectiveness is coded as 1 (low), 2 (moderate), or 3 (high); and evidence for ineffectiveness is represented by the corresponding negative values (-1, -2, -3).

To assess the *claimed* strength of the evidence communicated by each of the 100 health information webpages, we developed a parallel 7-point scale. This scale reflects commonly used lay terminology for different levels of evidence strength [23]:

-3: The webpage *expresses no doubts* about the intervention being ineffective.

-2: The webpage implies that the intervention is *probably or likely* ineffective.

-1: The webpage implies that the intervention *may or could be* ineffective.

0: The webpage suggests that it is unclear or insufficiently researched whether the intervention is effective.

1: The webpage implies that the intervention *may or could be* effective.

2: The webpage implies that the intervention is *probably or likely* effective.

3: The webpage *expresses no doubts* about the effectiveness of the intervention.

Two members of the research team (BK, IM) independently rated the *claimed* strength of evidence for each of the 100 health information webpages. Disagreements were solved through discussion to reach a consensus rating.

### Operationalization of factual correctness as a proxy for trustworthiness

As previously described [24], we operationalized the degree of factual correctness as the difference between the *factual* strength of evidence and the *claimed* strength of evidence, using it as a proxy for a webpage’s trustworthiness. Webpages were classified as containing incorrect information if this difference exceeded 1 (on a possible range of 0–6). Information was classified as correct when the difference was 0 or 1, allowing for minor uncertainty in the evidence ratings.

An exception applied when the *factual* strength of evidence was 0 (insufficient). In such cases, a *claimed* strength of evidence of 1 or -1 (“maybe effective” or “maybe ineffective”) was also classified as incorrect, reflecting the frequent promotion of complementary or alternative treatments as potentially effective despite lacking both proven efficacy and biological plausibility.

### Statistical analysis for predictive validity

The statistical analysis method was designed to answer the question: which items can best predict the factual correctness of a health claim on a webpage? We used the trustworthiness ratings of each of the 23 items from our application tests (see section “Application testing with research team members”) and the ratings on factual correctness for each webpage in the test set to calculate each item’s predictive validity.

To analyze the relationship between the factual correctness and the item ratings across specific items of interest, a Bayesian logistic regression model was employed and specified using the brms package in R (version 4.2.2) [25]. Data wrangling visualization was done via the tidyverse package [26].

The dependent variable, binary, indicated whether a given response was correct (1) or incorrect (0). We used the interaction between the rating (a continuous variable) and the ID_Item (a variable representing the individual items) as a predictor. This interaction term allows the effect of rating on the probability of a correct response to vary across different items.

The model was defined as follows:

correct∼1+rating:ID_Item

This formula indicates that the model includes an intercept and an interaction between the rating and the ID_Item. The outcome variable was modeled using a Bernoulli distribution. The prior for the intercept was a normal distribution with a mean of 0 and a standard deviation of 2 (normal(0, 2)). The prior for the regression coefficients (b) was also a normal distribution with a mean of 0 and a standard deviation of 2 (normal(0, 2)). These priors reflect weakly informative assumptions, allowing the data to primarily inform the posterior distributions.

The items were finally ordered according to their predictive validity.

### Creating the final checklist

We selected the final set of checklist items based on their predictive validity and several additional considerations. Alongside the regression coefficients for the predictive validity, we incorporated:

- laypersons’ judgments of the item difficulty and perceived importance,
- qualitative comments from lay users,
- qualitative notes from the research team regarding the item difficulty, importance, or ambiguity, and
- quantitative indicators of the item performance, including the interrater agreement among the laypersons, the agreement among the research team, the agreement between the laypersons and research team, and the mean difference between the expert and lay ratings.

These factors were jointly evaluated in the overall discussion to determine which items were included in the final checklist. This means that no single factor was decisive for inclusion in the final checklist; rather, decisions were based on the combined results, allowing for triangulation across the different inputs.

To support practical application, we also developed brief explanatory notes for each item and prepared a downloadable PDF containing the checklist and these explanations. For the purpose of this paper, both the items and the explanations were translated from German to English.

One team member drafted the initial translation, which was then reviewed and commented on by other members, and subsequently refined through discussion among the full research group.

# Results of the literature search

Table S2: Relevant reviews or pertinent articles, of which we checked the included tools and checklists if they are eligible to be included in the data extraction (n=11)

| **Reference type** | **Reference details** |
| --- | --- |
| Journal article | Abdel-Wahab N, Rai D, Siddhanamatha H, Dodeja A, Suarez-Almazor ME, Lopez-Olivo MA. A comprehensive scoping review to identify standards for the development of health information resources on the internet. PLoS ONE. 2019;14(6):e0218342. DOI: 10.1371/journal.pone.0218342. |
| Journal article | Bernstam EV, Shelton DM, Walji M, Meric-Bernstam F. Instruments to assess the quality of health information on the World Wide Web: what can our patients actually use? Int J Med Inform. 2005;74(1):13-9. DOI: 10.1016/j.ijmedinf.2004.10.001. |
| Journal article | Daraz L, Morrow AS, Ponce OJ, Beuschel B, Farah MH, Katabi A, et al. Can Patients Trust Online Health Information? A Meta-narrative Systematic Review Addressing the Quality of Health Information on the Internet. J Gen Intern Med. 2019;34(9):1884-91. DOI: 10.1007/s11606-019-05109-0. |
| Journal article | Dobbins M, Watson S, Read K, Graham K, Yousefi Nooraie R, Levinson AJ. A Tool That Assesses the Evidence, Transparency, and Usability of Online Health Information: Development and Reliability Assessment. JMIR Aging. 2018;1(1):e3. DOI: 10.2196/aging.9216. |
| Journal article | Hanif F, Read JC, Goodacre JA, Chaudhry A, Gibbs P. The role of quality tools in assessing reliability of the internet for health information. Inform Health Soc Care. 2009;34(4):231-43. DOI: 10.3109/17538150903359030. |
| Journal article | Lenaerts G, Bekkering GE, Goossens M, De Coninck L, Delvaux N, Cordyn S, et al. Tools to Assess the Trustworthiness of Evidence-Based Point-of-Care Information for Health Care Professionals: Systematic Review. J Med Internet Res. 2020;22(1):e15415. DOI: 10.2196/15415. |
| Journal article | Risk A, Dzenowagis J. Review of internet health information quality initiatives. J Med Internet Res. 2001;3(4):E28. DOI: 10.2196/jmir.3.4.e28. |
| Journal article | Robillard JM, Jun JH, Lai JA, Feng TL. The QUEST for quality online health information: validation of a short quantitative tool. BMC Med Inform Decis Mak. 2018;18(1):87. DOI: 10.1186/s12911-018-0668-9. |
| Report | Siebenhofer A, Posch N. MAPPinfo (MAPPing quality of health INFOrmation) – Checkliste Version 1.1 2021. |
| Journal article | Song S, Zhang Y, Yu B. Interventions to support consumer evaluation of online health information credibility: A scoping review. Int J Med Informatics. 2021;145:104321. DOI: 10.1016/j.ijmedinf.2020.104321. |
| Journal article | Zhang Y, Sun Y, Xie B. Quality of health information for consumers on the web: A systematic review of indicators, criteria, tools, and evaluation results. J Assoc Soc Inf Sci Technol. 2015;66(10):2071-84. DOI: 10.1002/asi.23311. |

Table S3: Tools and checklists included for data extraction (n=46)

| **Reference type** | **Reference details** |
| --- | --- |
| Journal article | Blakeslee S. The CRAAP Test. LOEX Quarterly. 2004;31(3):6-7. |
| Journal article | Boyer C, Baujard V, Geissbuhler A. Evolution of health web certification through the HONcode experience. Stud Health Technol Inform. 2011;169:53-7. |
| Journal article | Charvet-Berard AI, Chopard P, Perneger TV. Measuring quality of patient information documents with an expanded EQIP scale. Patient Educ Couns. 2008;70(3):407-11. DOI: 10.1016/j.pec.2007.11.018. |
| Journal article | Childs S. Judging the quality of internet‐based health information. Performance Measurement and Metrics. 2005;6(2):80-96. DOI: 10.1108/14678040510607803. |
| Journal article | Dai E, Sun Y, Wang S. Ginger Cannot Cure Cancer: Battling Fake Health News with a Comprehensive Data Repository. arXivorg. 2020. DOI: 10.5281/zenodo.3606757. |
| Journal article | Daraz L, Morrow AS, Ponce OJ, Beuschel B, Farah MH, Katabi A, et al. Can Patients Trust Online Health Information? A Meta-narrative Systematic Review Addressing the Quality of Health Information on the Internet. J Gen Intern Med. 2019;34(9):1884-91. DOI: 10.1007/s11606-019-05109-0. |
| Journal article | Dobbins M, Watson S, Read K, Graham K, Yousefi Nooraie R, Levinson AJ. A Tool That Assesses the Evidence, Transparency, and Usability of Online Health Information: Development and Reliability Assessment. JMIR Aging. 2018;1(1):e3. DOI: 10.2196/aging.9216. |
| Journal article | Johnson ST, Ewbank AD. Heuristics: An Approach to Evaluating News Obtained through Social Media. Knowledge Quest. 2018;47(1). |
| Journal article | Kurtz-Rossi S, Duguay P. Health Information Literacy Outreach: Improving Health Literacy and Access to Reliable Health Information in Rural Oxford County Maine. Journal of Consumer Health on the Internet. 2010;14(4):325-40. DOI: 10.1080/15397734.2010.524089. |
| Journal article | Mandalios J. RADAR: An approach for helping students evaluate Internet sources. Journal of Information Science. 2013;39(4):470-8. DOI: 10.1177/0165551513478889. |
| Journal article | Narhi U, Pohjanoksa-Mantyla M, Karjalainen A, Saari JK, Wahlroos H, Airaksinen MS, et al. The DARTS tool for assessing online medicines information. Pharm World Sci. 2008;30(6):898-906. DOI: 10.1007/s11096-008-9249-9. |
| Journal article | Provost M, Koompalum D, Dong D, Martin BC. The initial development of the WebMedQual scale: domain assessment of the construct of quality of health web sites. Int J Med Inform. 2006;75(1):42-57. DOI: 10.1016/j.ijmedinf.2005.07.034. |
| Journal article | Roberts L. Health information and the Internet: The 5 Cs website evaluation tool. Br J Nurs. 2010;19(5):322-5. DOI: 10.12968/bjon.2010.19.5.47075. |
| Journal article | Robillard JM, Jun JH, Lai JA, Feng TL. The QUEST for quality online health information: validation of a short quantitative tool. BMC Med Inform Decis Mak. 2018;18(1):87. DOI: 10.1186/s12911-018-0668-9. |
| Journal article | Russo A, Jankowski A, Beene S, Townsend L. Strategic source evaluation: addressing the container conundrum. Reference Services Review. 2019;47(3):294-313. DOI: 10.1108/rsr-04-2019-0024. |
| Journal article | Winker MA, Flanagin A, Chi-Lum B, White J, Andrews K, Kennett RL, et al. Guidelines for medical and health information sites on the internet: principles governing AMA web sites. American Medical Association. Jama. 2000;283(12):1600-6. DOI: 10.1001/jama.283.12.1600. |
| Book or book section | Ballstaedt SP. Kapitel 11: Texte evaluieren und optimieren. Sprachliche Kommunikation: Verstehen und Verständlichkeit. Tübingen: Narr Francke Attempto Verlag; 2019. |
| Book or book section | Charnock D. The DISCERN Handbook. Quality criteria for consumer health information on treatment choices. Abingdon: University of Oxford and The British Library; 1998. |
| Book or book section | Hölling G, Schmidt H, Thelen M. gesundheitsziele.de: Qualitätskriterien für Gesundheitsinformationen – Eine Checkliste für Bürger/innen und Patient(inn)en. In: Gesellschaft für Versicherungswissenschaft und -gestaltung e.V. (GVG), editor. Gesundheitsinformationen in Deutschland Eine Übersicht zu Anforderungen, Angeboten und Herausforderungen. Köln2011. p. 31-6. |
| Report | Bertelsmann Stiftung. Kriterien zur Beurteilung des Schadenspotenzials von Gesundheitsinformationen: <https://www.bertelsmann-stiftung.de/fileadmin/files/Projekte/Patient_mit_Wirkung/VV_Kriterienraster_digital_final.pdf> (Accessed: 13 Oct 2021). |
| Report | ÖPGK. Gute Gesundheitsinformation Österreich. Die 15 Qualitätskriterien. Der Weg zum Methodenpapier — Anleitung für Organisationen: <https://oepgk.at/wp-content/uploads/2020/12/2020_11_18_die-gute-gesundheitsinformation.pdf> (Accessed: 05 Oct 2021). |
| Report | Österreichische Plattform Gesundheitskompetenz (ÖPGK). Verständliche Sprache beim Verfassen von schriftlichen Gesundheitsinformationen. ÖPGK-Factsheet. Wien: ÖPGK; 2020. Available from: <https://oepgk.at/wp-content/uploads/2020/10/oepgk_factsheet_leichte_sprache_bfrei.pdf> |
| Report | Siebenhofer A, Posch N. MAPPinfo (MAPPing quality of health INFOrmation) – Checkliste Version 1.1 2021. |
| Webpage | Aktionsforum Gesundheitsinformationssystem (afgis). Ein Leitfaden für die Erstellung des eigenen Webangebots: <https://www.afgis.de/standards/medizinische-websites/> (Accessed: 21 Oct 2021). |
| Webpage | Checkliste zur Bewertung von Internetquellen für Schülerinnen und Schüler: <https://li.hamburg.de/contentblob/3461588/aeeb63b90b0c1ca82dbb0737d318392c/data/pdf-internetquellen-bewerten-in-der-profiloberstufe.pdf> (Accessed: 02 Nov 2021). |
| Webpage | Deutsches Netzwerk Gesundheitskompetenz DNGK - Health Literacy Network Germany. Verlässliches Gesundheitswissen: <https://dngk.de/verlaessliches-gesundheitswissen/> (Accessed: 04 Nov 2021). |
| Webpage | Faktencheck-Gesundheitswerbung.de. Checkliste für gute Gesundheitsinformation: <https://www.faktencheck-gesundheitswerbung.de/gesund-im-netz/checkliste-fuer-gute-gesundheitsinformation-54474> (Accessed: 03 Nov 2021). |
| Webpage | Feekery A, Jeffrey C, Kara SMH. Rauru Whakarare Evaluation Framework: <https://informationliteracyspaces.files.wordpress.com/2019/07/rauru-whakarare-framework-and-descriptors-2.pdf> (Accessed: 03 Nov 2021). |
| Webpage | Frauengesundheitszentrum. Checkliste zur Bewertung von Gesundheitsinformation: <http://www.frauengesundheitszentrum.eu/wp-content/uploads/2016/09/Checkliste-Gesundheitsinformation_Frauengesundheitszentrum_032019.pdf> (Accessed: 03 Nov 2021). |
| Webpage | Gesund informiert entscheiden. Gute Gesundheitsinformationen: <https://www.gesund-informiert.at/gesundheitsinformation/gute-gesundheitsinformationen> (Accessed: 23 Sept 2021). |
| Webpage | Gesund-im-netz.net. Allgemeine Suchtipps: <https://www.gesund-im-netz.net/wegweiser/suchtipps/> (Accessed: 05 Oct 2021). |
| Webpage | Gesund-im-netz.net. Unsere Auswahlkriterien: <https://www.gesund-im-netz.net/wegweiser/linkliste/unsere-auswahlkritertien/> (Accessed: 21 Oct 2021). |
| Webpage | GÖG/BIQG. Checkliste Kriterien für die Erstellung von Patienteninformation <https://jasmin.goeg.at/1794/2/Checkliste%20-%20Erstellung%20von%20%20Informationsmaterial.pdf> (Accessed: 05 Oct 2021). |
| Webpage | Guertin L, Clements N. CRAP test for information literacy: <https://scholarsphere.psu.edu/resources/76c558da-b7f9-4704-a7b1-54559d0bdd20> (Accessed: 06 Sept 2021). |
| Webpage | Institut für Qualität und Transparenz von Gesundheitsinformationen. Broschüre "Gute Gesundheitsinformationen im Netz finden und erkennen": <https://www.iqtg.de/cms/index.asp?inst=iqtg&snr=8957&t=%22gute+Gesundheitsinformationen+finden+und+erkennen%22> (Accessed: 20 Oct 2021). |
| Webpage | IQWiG. Wie finde ich gute Gesundheitsinformationen im Internet?: <https://www.gesundheitsinformation.de/pdf/informationsmaterialien/flyer-gute-gi.pdf?rev=122486> (Accessed: 20 Oct 2021). |
| Webpage | Medizin-Transparent.at. Checkliste: Gesundheitsmythen - Fake news erkennen: <https://www.medizin-transparent.at/ueber/gesundheitsmythen-fake-news-erkennen/> (Accessed: 15 Sept 2021). |
| Webpage | Minervation. The LIDA Instrument: <https://www.minervation.com/wp-content/uploads/2011/04/Minervation-LIDA-instrument-v1-2.pdf> |
| Webpage | National Institute on Aging. Welcome to Module 9: Evaluating Health Websites: <www.nihseniorhealth.gov/toolkit> (Accessed: 03 Nov 2021). |
| Webpage | NHS. The Information Standard Principles: <https://www.england.nhs.uk/tis/about/the-info-standard/> (Accessed: 20 Sept 2021). |
| Webpage | ÖPGK. Checkliste Gute Gesundheitsinformation, Version 1.0, April 2020: <https://oepgk.at/wp-content/uploads/2020/10/oepgk_ggi_allg_checkliste_bfrei.pdf> (Accessed: 05 Oct 2021). |
| Webpage | ÖPGK. Überblick über die 15 Qualitätskriterien für zielgruppenorientierte, evidenzbasierte Broschüren, Videos, Websites und Apps: <https://oepgk.at/wp-content/uploads/2020/12/2020_11_18_fuenfzehn-qualitaetskriterien.pdf> (Accessed: 05 Oct 2021). |
| Webpage | Patienten Universität. Tipps für die Suche im Internet: <https://www.patienten-universitaet.de/content/tipps-f%C3%BCr-die-suche-im-internet> (Accessed: 06 Oct 2021). |
| Webpage | Patienten-Information.de - Gut informiert entscheiden. Gute Informationen im Netz finden: [https://www.patienten-information.de/kurzinformationen/gesundheitsthemen-im-internet#](https://www.patienten-information.de/kurzinformationen/gesundheitsthemen-im-internet) (Accessed: 07 Oct 2021). |
| Webpage | Patienten-Information.de - Gut informiert entscheiden. Neuer Patientenfilm: Gesundheitsinformationen im Internet: <https://www.patienten-information.de/news/neue-aezq-gesundheitsinformation-2020-07-02> (Accessed: 07 Oct 2021). |
| Webpage | Universitätsklinikum Freiburg. So entlarven Sie medizinische Fake News. Gesundheitstipps: <https://www.uniklinik-freiburg.de/presse/publikationen/im-fokus/so-entlarven-sie-medizinische-fake-news.html> (Accessed: 02 Nov 2021). |

Table S4: Literature on concepts, quality criteria, and indicators relevant to evaluating online health information included for data extraction (n=27)

| **Reference type** | **Reference details** |
| --- | --- |
| Journal article | Abdel-Wahab N, Rai D, Siddhanamatha H, Dodeja A, Suarez-Almazor ME, Lopez-Olivo MA. A comprehensive scoping review to identify standards for the development of health information resources on the internet. PLoS ONE. 2019;14(6):e0218342. DOI: 10.1371/journal.pone.0218342. |
| Journal article | Bernstam EV, Shelton DM, Walji M, Meric-Bernstam F. Instruments to assess the quality of health information on the World Wide Web: what can our patients actually use? Int J Med Inform. 2005;74(1):13-9. DOI: 10.1016/j.ijmedinf.2004.10.001. |
| Journal article | Bunge M, Muhlhauser I, Steckelberg A. What constitutes evidence-based patient information? Overview of discussed criteria. Patient Educ Couns. 2010;78(3):316-28. DOI: 10.1016/j.pec.2009.10.029. |
| Journal article | Chalmers I, Oxman AD, Austvoll-Dahlgren A, Ryan-Vig S, Pannell S, Sewankambo N, et al. Key Concepts for Informed Health Choices: a framework for helping people learn how to assess treatment claims and make informed choices. BMJ Evidence-Based Medicine. 2018;23(1):29-33. DOI: 10.1136/ebmed-2017-110829. |
| Journal article | Corritore CL, Wiedenbeck S, Kracher B, Marble RP. Online Trust and Health Information Websites. International Journal of Technology and Human Interaction. 2012;8(4):92-115. DOI: 10.4018/jthi.2012100106. |
| Journal article | Elmwood V. The Journalistic Approach: Evaluating Web Sources in an Age of Mass Disinformation. Communications in Information Literacy. 2020;14(2). DOI: 10.15760/comminfolit.2020.14.2.6. |
| Journal article | Eysenbach G, Powell J, Kuss O, Sa ER. Empirical studies assessing the quality of health information for consumers on the World Wide Web: A systematic review. J Am Med Assoc. 2002;287(20):2691-700. DOI: 10.1001/jama.287.20.2691. |
| Journal article | Johnson F, Rowley J, Sbaffi L. Modelling trust formation in health information contexts. Journal of Information Science. 2015;41(4):415-29. DOI: 10.1177/0165551515577914. |
| Journal article | Keselman A, Arnott Smith C, Murcko AC, Kaufman DR. Evaluating the Quality of Health Information in a Changing Digital Ecosystem. J Med Internet Res. 2019;21(2):e11129. DOI: 10.2196/11129. |
| Journal article | Kim Y. Trust in health information websites: A systematic literature review on the antecedents of trust. Health Informatics J. 2016;22(2):355-69. DOI: 10.1177/1460458214559432. |
| Journal article | Konig L, Jucks R. Influence of Enthusiastic Language on the Credibility of Health Information and the Trustworthiness of Science Communicators: Insights From a Between-Subject Web-Based Experiment. Interact J Med Res. 2019;8(3):e13619. DOI: 10.2196/13619. |
| Journal article | Lovett J, Gordon C, Patton S, Chen CX. Online information on dysmenorrhoea: An evaluation of readability, credibility, quality and usability. J Clin Nurs. 2019;28(19-20):3590-8. DOI: 10.1111/jocn.14954. |
| Journal article | O'Grady L. Future directions for depicting credibility in health care web sites. Int J Med Inform. 2006;75(1):58-65. DOI: 10.1016/j.ijmedinf.2005.07.035. |
| Journal article | Ostenson J. Reconsidering the Checklist in Teaching Internet Source Evaluation. portal: Libraries and the Academy. 2013;14(1):33-50. DOI: 10.1353/pla.2013.0045. |
| Journal article | Sandvik H. Health information and interaction on the internet: a survey of female urinary incontinence. Bmj. 1999;319(7201):29-32. DOI: 10.1136/bmj.319.7201.29. |
| Journal article | Sbaffi L, Rowley J. Trust and Credibility in Web-Based Health Information: A Review and Agenda for Future Research. J Med Internet Res. 2017;19(6):e218. DOI: 10.2196/jmir.7579. |
| Journal article | Silberg WM, Lundberg GD, Musacchio RA. Assessing, controlling, and assuring the quality of medical information on the Internet: Caveant lector et viewor--Let the reader and viewer beware. Jama. 1997;277(15):1244-5. |
| Journal article | Sun Y, Zhang Y, Gwizdka J, Trace CB. Consumer Evaluation of the Quality of Online Health Information: Systematic Literature Review of Relevant Criteria and Indicators. J Med Internet Res. 2019;21(5):e12522. DOI: 10.2196/12522. |
| Journal article | Thon FM, Jucks R. Believing in Expertise: How Authors' Credentials and Language Use Influence the Credibility of Online Health Information. Health Commun. 2017;32(7):828-36. DOI: 10.1080/10410236.2016.1172296. |
| Journal article | Ting DK, Boreskie P, Luckett-Gatopoulos S, Gysel L, Lanktree MB, Chan TM. Quality Appraisal and Assurance Techniques for Free Open Access Medical Education (FOAM) Resources: A Rapid Review. Semin Nephrol. 2020;40(3):309-19. DOI: 10.1016/j.semnephrol.2020.04.011. |
| Journal article | Zhang Y, Sun Y, Xie B. Quality of health information for consumers on the web: A systematic review of indicators, criteria, tools, and evaluation results. J Assoc Soc Inf Sci Technol. 2015;66(10):2071-84. DOI: 10.1002/asi.23311. |
| Conference paper | Vervier L, Ziefle ACVM. “Should I Trust or Should I Go?” or What Makes Health-Related Websites Appear Trustworthy? 4th International Conference on Information and Communication Technologies for Ageing Well and e-Health2018. |
| Conference paper | Zhang AX, Ranganathan A, Metz SE, Appling S, Sehat CM, Gilmore N, et al. A Structured Response to Misinformation: Defining and Annotating Credibility Indicators in News Articles. Companion Proceedings of the The Web Conference 2018; Lyon, France: International World Wide Web Conferences Steering Committee; 2018. p. 603–12. DOI: 10.1145/3184558.3188731. |
| Webpage | Informed Health Choices. Key Concepts. A framework for developing and identifying learning resources: <https://www.informedhealthchoices.org/key-concepts-2-2/> (Accessed: 20 Oct 2021). |
| Webpage | KBV. Handbuch Qualitätszirkel - 4.7. Patienteninformation. Moderatorenfortbildung: <https://www.kbv.de/media/sp/4.7_Patienteninformation.pdf> (Accessed: 20 Oct 2021). |
| Webpage | Lühnen J, Albrecht M, Mühlhauser I, Steckelberg A. Leitlinie evidenzbasierte Gesundheitsinformation: <http://www.leitlinie-gesundheitsinformation.de/> (Accessed: 20 Oct 2021). |
| Webpage | NÖ Patienten- und Pflegeanwaltschaft. Wie finde ich seriöse Gesundheitsinformation im Internet? Praxisleitfaden: <https://www.patientenanwalt.com/wp-content/uploads/2014/01/Praxisleitfaden_Internet_Broschuere1.pdf> (Accessed: 20 Sept 2021). |

# Interview guide used in the cognitive interviews

# Supplementary tables for the results of the Delphi process

Table S5: Overview of the results of Delphi round 1 for items for evaluating health information on websites (number of items to be rated = 46), ratings for appropriateness and applicability

|  |  | **Appropriateness ratings** | | | | **Applicability ratings** | | | |
| --- | --- | --- | --- | --- | --- | --- | --- | --- | --- |
|  | **# items** | **M of M ratings** | **M of SD** | **Min of Min** | **Max of Max** | **M of M ratings** | **M of SD** | **Min of Min** | **Max of Max** |
| D.1 Comprehensibility | 1 | 3.33 | 1.25 | 1 | 5 | 1.67 | 0.47 | 1 | 2 |
| D.2 Layout | 1 | 3.80 | 1.57 | 0 | 5 | 4.80 | 1.83 | 0 | 5 |
| D.3 Linguistic style | 4 | 4.43 | 0.78 | 0 | 5 | 3.66 | 0.84 | 0 | 5 |
| I.1. Balance | 13 | 4.53 | 0.83 | 0 | 5 | 3.50 | 1.23 | 0 | 5 |
| I.2 References | 3 | 4.61 | 1.24 | 0 | 5 | 3.03 | 1.19 | 0 | 5 |
| I.3 Level of evidence | 2 | 4.58 | 0.74 | 2 | 5 | 3.25 | 1.00 | 2 | 5 |
| I.4 Quality assurance | 1 | 3.83 | 1.46 | 1 | 5 | 2.83 | 1.46 | 1 | 5 |
| I.5 Methods | 3 | 4.44 | 0.91 | 2 | 5 | 3.22 | 1.16 | 1 | 5 |
| I.6 Further information | 1 | 4.33 | 1.11 | 2 | 5 | 5.00 | 0.00 | 5 | 5 |
| N.1 Emotion | 1 | 4.33 | 0.75 | 3 | 5 | 4.17 | 0.90 | 3 | 5 |
| N.2 Familiarity and reputation | 1 | 3.67 | 1.60 | 1 | 5 | 2.33 | 0.94 | 1 | 4 |
| N.3 Trustworthiness of content | 3 | 3.83 | 1.46 | 0 | 5 | 3.17 | 1.06 | 0 | 5 |
| N.4 Trustworthiness of references | 1 | 4.50 | 0.76 | 3 | 5 | 3.83 | 1.21 | 2 | 5 |
| N.5 Trustworthiness in general | 2 | 3.83 | 1.10 | 1 | 5 | 3.83 | 1.21 | 1 | 5 |
| T.1 Up-to-date information | 2 | 4.92 | 0.19 | 4 | 5 | 4.83 | 0.37 | 3 | 5 |
| T.2 Author and copyright | 2 | 4.83 | 0.24 | 4 | 5 | 3.92 | 1.00 | 2 | 5 |
| T.4 Disclosure and funding | 4 | 4.71 | 0.56 | 3 | 5 | 3.88 | 0.79 | 2 | 5 |
| T.5 Standards/certificates | 1 | 4.00 | 0.82 | 3 | 5 | 4.17 | 1.07 | 2 | 5 |

Note: A min value of 0 appears due to missing values.

Abbreviations: M, mean; Max, maximum; Min, minimum; SD, standard deviation; T, transparency; I, presentation of information; D, presentation (linguistic and visual); N, user perception

Table S6: Overview of the results of Delphi round 2 for items for evaluating health information on websites (number of items to be rated = 52), ratings for appropriateness and applicability

|  |  | **Appropriateness ratings** | | | | **Applicability ratings** | | | |
| --- | --- | --- | --- | --- | --- | --- | --- | --- | --- |
|  | **# items** | **M of M ratings** | **M of SD** | **Min of Min** | **Max of Max** | **M of M ratings** | **M of SD** | **Min of Min** | **Max of Max** |
| D.1 Comprehensibility | 1 | 3.33 | 1.25 | 1 | 5 | 1.67 | 0.47 | 1 | 2 |
| D.2 Layout | 1 | 3.80 | 1.57 | 0 | 5 | 4.80 | 1.83 | 0 | 5 |
| D.3 Linguistic style | 4 | 4.43 | 0.78 | 0 | 5 | 3.66 | 0.84 | 0 | 5 |
| I.1. Balance | 13 | 4.53 | 0.83 | 0 | 5 | 3.50 | 1.23 | 0 | 5 |
| I.2 References | 3 | 4.61 | 1.24 | 0 | 5 | 3.03 | 1.19 | 0 | 5 |
| I.3 Level of evidence | 2 | 4.58 | 0.74 | 2 | 5 | 3.25 | 1.00 | 2 | 5 |
| I.4 Quality assurance | 1 | 3.83 | 1.46 | 1 | 5 | 2.83 | 1.46 | 1 | 5 |
| I.5 Methods | 3 | 4.44 | 0.91 | 2 | 5 | 3.22 | 1.16 | 1 | 5 |
| I.6 Further information | 1 | 4.33 | 1.11 | 2 | 5 | 5.00 | 0.00 | 5 | 5 |
| N.1 Emotion | 1 | 4.33 | 0.75 | 3 | 5 | 4.17 | 0.90 | 3 | 5 |
| N.2 Familiarity and reputation | 1 | 3.67 | 1.60 | 1 | 5 | 2.33 | 0.94 | 1 | 4 |
| N.3 Trustworthiness of content | 3 | 3.83 | 1.46 | 0 | 5 | 3.17 | 1.06 | 0 | 5 |
| N.4 Trustworthiness of references | 1 | 4.50 | 0.76 | 3 | 5 | 3.83 | 1.21 | 2 | 5 |
| N.5 Trustworthiness in general | 2 | 3.83 | 1.10 | 1 | 5 | 3.83 | 1.21 | 1 | 5 |
| T.1 Up-to-date information | 2 | 4.92 | 0.19 | 4 | 5 | 4.83 | 0.37 | 3 | 5 |
| T.2 Author and copyright | 2 | 4.83 | 0.24 | 4 | 5 | 3.92 | 1.00 | 2 | 5 |
| T.4 Disclosure and funding | 4 | 4.71 | 0.56 | 3 | 5 | 3.88 | 0.79 | 2 | 5 |
| T.5 Standards/certificates | 1 | 4.00 | 0.82 | 3 | 5 | 4.17 | 1.07 | 2 | 5 |

Note: Min value of 0 appears due to missing values.

Abbreviations: M, mean; Max, maximum; Min, minimum; SD, standard deviation; T, transparency; I, presentation of information; D, presentation (linguistic and visual); N, user perception

Table S7: Differences between item ratings in the expert Delphi rounds (Round 2 - Round 1)

|  | **# items** | **Diff Round 2 - Round 1 for appropriateness** | **Diff Round 2 - Round 1 for applicability** |
| --- | --- | --- | --- |
| D.1 Comprehensibility | 1 | -0.13 | 0.53 |
| D.2 Layout | 1 | -0.13 | -0.63 |
| D.3 Linguistic style | 4 | -0.22 | 0.01 |
| I.1 Balance | 13 | -0.08 | 0.17 |
| I.2 References | 3 | 0.00 | 0.08 |
| I.3 Level of evidence | 2 | 0.17 | 0.42 |
| I.4 Quality assurance | 1 | 0.17 | 0.33 |
| I.5 Methods | 3 | 0.11 | 0.28 |
| I.6 Further information | 1 | 0.00 | -0.33 |
| N.1 Emotion | 1 | 0.17 | 0.00 |
| N.2 Familiarity and reputation | 1 | 0.50 | 0.50 |
| N.3 Trustworthiness of content | 3 | 0.28 | 0.33 |
| N.4 Trustworthiness of references | 1 | 0.50 | -0.17 |
| N.5 Trustworthiness in general | 2 | 0.25 | 0.08 |
| T.1 Up-to-date information | 2 | -0.17 | -0.25 |
| T.2 Author and copyright | 2 | -0.17 | 0.33 |
| T.4 Disclosure and funding | 4 | -0.08 | -0.21 |
| T.5 Standards/certificates | 1 | 0.33 | 0.17 |

Abbreviations: Diff, Difference

# Test set of health information webpages

Table S8: 100 Health information webpages in the German language used as a test set for the iWISE checklist validation

|  | **Topic** | **Web portal** | **URL** |
| --- | --- | --- | --- |
| 1 | Does vitamin C help with a cold? | Gesundheitsinformation.de | https://www.gesundheitsinformation.de/schnupfen-husten-und-halsschmerzen-lindern.html |
| 2 | Does vitamin C help with a cold? | Herbano.com | https://herbano.com/at/ratgeber/viren-erkaeltung-stoppen |
| 3 | Does vitamin C help with a cold? | Stiftung-Gesundheitswissen.de | https://www.stiftung-gesundheitswissen.de/gesundes-leben/koerper-wissen/mutti-oder-medizin-helfen-hausmittel-bei-erkaeltung#vitamin-c-bei-erkaeltungen |
| 4 | Does vitamin C help with a cold? | Zentrum-der-gesundheit.de | https://www.zentrum-der-gesundheit.de/news/gesundheit/allgemein-gesundheit/nahrungsergaenzungsmittel-erkaeltung |
| 5 | Does vitamin C help with a cold? | My-health.ch | https://www.my-health.ch/erkaeltung-behandeln-mit-naehrstoffen-von-echinacea-bis-zink/ |
| 6 | Does vitamin C help with a cold? | Dualdiagnosis.org | https://dualdiagnosis.org/de/ratgeber/zink-bei-erkaeltung/ |
| 7 | Does vitamin C help with a cold? | Sozialversicherung.at | https://www.sozialversicherung.at/cdscontent/?contentid=10007.862784&portal=svportal |
| 8 | Does vitamin C help with a cold? | medizinfuchs.de | https://www.medizinfuchs.de/blog/leben/medizinfuchs-mythen/der-schlaue-fuchs-hilft-vitamin-c-bei-erkaeltungen/ |
| 9 | Does vitamin C help with a cold? | Pharmazeutische-Zeitung.de | https://www.pharmazeutische-zeitung.de/ausgabe-482015/vitamin-c-bei-erkaeltungen/ |
| 10 | Does vitamin C help with a cold? | GU.de | https://www.gu.de/hilft-vitamin-c-bei-erkaeltung/ |
| 11 | Do cranberries help with bladder infection? | Dr-boehm.at | https://www.dr-boehm.at/ratgeber/beerenstark-die-cranberry-bei-harnwegsinfekt/ |
| 12 | Do cranberries help with bladder infection? | aerzteblatt.de | https://www.aerzteblatt.de/nachrichten/128011/Cranberry-statt-Antibiotika-Alternative-Behandlungsoption-bei-Blasenentzuendung |
| 13 | Do cranberries help with bladder infection? | klartext-nahrungsergaenzung.de | https://www.klartext-nahrungsergaenzung.de/wissen/lebensmittel/nahrungsergaenzungsmittel/cranberry-sind-produkte-zur-vorbeugung-von-blasenentzuendungen-geeignet-8143 |
| 14 | Do cranberries help with bladder infection? | Mylife.de | https://www.mylife.de/heilpflanzen/cranberry/ |
| 15 | Do cranberries help with bladder infection? | movement21.at | https://www.movement21.at/imindmyfood/rubriken/ernaehrung/wirksame-vorbeugung-gegen-blasenentzuendung-mit-der-powerbeere-cranberry |
| 16 | Do cranberries help with bladder infection? | gesundheit.de | https://www.gesundheit.de/ernaehrung/lebensmittel/obst/cranberry |
| 17 | Do cranberries help with bladder infection? | Onmeda.de | https://www.onmeda.de/krankheiten/blasenentzuendung/cranberrysaft-id203042/ |
| 18 | Do cranberries help with bladder infection? | Phytodoc.de | https://www.phytodoc.de/heilpflanzen/cranberry |
| 19 | Do cranberries help with bladder infection? | Medicom.de | https://www.medicom.de/wirkstoffe/pflanzenstoffe/cranberry |
| 20 | Do cranberries help with bladder infection? | Infosperber.ch | https://www.infosperber.ch/gesundheit/wiederkehrende-blasenentzuendung-cranberry-praeparate-nuetzen/ |
| 21 | Does arthroscopy help with osteoarthritis? | netdoktor.at | https://www.netdoktor.at/krankheiten/arthrose/ |
| 22 | Does arthroscopy help with osteoarthritis? | Gelenke-brauchen-mehr.de | https://www.gelenke-brauchen-mehr.de/arthrose/diagnose/arthroskopie |
| 23 | Does arthroscopy help with osteoarthritis? | Gesundheitsinformation.de | https://www.gesundheitsinformation.de/hilft-eine-arthroskopie-bei-kniearthrose.html |
| 24 | Does arthroscopy help with osteoarthritis? | Pyramide.ch | https://pyramide.ch/de/gelenk-und-sportchirurgie/knieoperationen/knie-arthroskopie/ |
| 25 | Does arthroscopy help with osteoarthritis? | Lifeline.de | https://www.lifeline.de/krankheiten/arthrose/kniespiegelung-gegen-arthrose-ist-nutzlos-id130722.html |
| 26 | Does arthroscopy help with osteoarthritis? | on-orthopaedie.de | https://www.on-orthopaedie.de/spektrum/arthroskopie-knie/kniearthrose/#operativ |
| 27 | Does arthroscopy help with osteoarthritis? | Gelenkexperten.com | https://gelenkexperten.com/operationen/arthroskopie/ |
| 28 | Does arthroscopy help with osteoarthritis? | Knorpelexperte.de | https://knorpelexperte.de/kniespiegelung/ |
| 29 | Does arthroscopy help with osteoarthritis? | apotheken-umschau.de | https://www.apotheken-umschau.de/therapie/therapiearten/arthroskopie-spiegelung-des-gelenks-742681.html |
| 30 | Does arthroscopy help with osteoarthritis? | theaurora.at | https://www.theaurora.at/leistungen/arthroskopie/ |
| 31 | Does ginkgo help with dementia? | Gesundheitsinformation.de | https://www.gesundheitsinformation.de/nuetzen-ginkgohaltige-mittel.html |
| 32 | Does ginkgo help with dementia? | Stiftung-Gesundheitswissen.de | https://www.stiftung-gesundheitswissen.de/wissen/alzheimer-demenz/behandlung |
| 33 | Does ginkgo help with dementia? | aerztezeitung.de | https://www.aerztezeitung.de/Medizin/Ginkgo-biloba-wirkt-auch-bei-Demenz-301830.html |
| 34 | Does ginkgo help with dementia? | demenz-portal.at | https://demenz-portal.at/aktuelles/ginkgo-in-der-forschung/ |
| 35 | Does ginkgo help with dementia? | medmix.at | https://medmix.at/ginkgo-gegen-demenz/ |
| 36 | Does ginkgo help with dementia? | alzheimer.ch | https://alzheimer.ch/magazin/wissen/forschung/sanftes-heilmittel-furs-gehirn/ |
| 37 | Does ginkgo help with dementia? | Mylife.de | https://www.mylife.de/heilpflanzen/ginkgo/ |
| 38 | Does ginkgo help with dementia? | medmix.at | https://medmix.at/ginkgo-biloba-wirkungen/ |
| 39 | Does ginkgo help with dementia? | vitaminexpress.org | https://www.vitaminexpress.org/at/ginkgo-biloba |
| 40 | Does ginkgo help with dementia? | apotheken-umschau.de | https://www.apotheken-umschau.de/medikamente/heilpflanzen/ginkgo-gut-fuer-das-gedaechtnis-736221.html |
| 41 | Does mistletoe therapy help with cancer? | misteltherapie.at | https://www.misteltherapie.at/die-misteltherapie/wirkung |
| 42 | Does mistletoe therapy help with cancer? | mistel-therapie.de | https://www.mistel-therapie.de/informationen-fuer-patientinnen |
| 43 | Does mistletoe therapy help with cancer? | krebsinformationsdienst.de | https://www.krebsinformationsdienst.de/behandlung/unkonv-methoden/mistel.php |
| 44 | Does mistletoe therapy help with cancer? | healthcare-in-europe.com | https://healthcare-in-europe.com/de/news/misteln-wirksamkeit-gegen-krebs-zweifelhaft.html |
| 45 | Does mistletoe therapy help with cancer? | integrative-onkologie.com | https://integrative-onkologie.com/leistungsspektrum/misteltherapie/ |
| 46 | Does mistletoe therapy help with cancer? | ordination-url.at | https://ordination-url.at/krebsbehandlungen/misteltherapie/ |
| 47 | Does mistletoe therapy help with cancer? | minimed.at | https://www.minimed.at/medizinische-themen/krebs/mistel-heilpflanze/ |
| 48 | Does mistletoe therapy help with cancer? | meine-gesundheit.de | https://www.meine-gesundheit.de/medizin/heilpflanzen/mistel |
| 49 | Does mistletoe therapy help with cancer? | biokrebs.de | https://www.biokrebs.de/therapien/immunsystem/mistel |
| 50 | Does mistletoe therapy help with cancer? | helixor.de | https://www.helixor.de/misteltherapie/ |
| 51 | Does a ketogenic diet help with cancer? | ugb.de | https://www.ugb.de/ernaehrungsberatung/ketogene-diaet-ernaehrung-bei-krebs/ |
| 52 | Does a ketogenic diet help with cancer? | heilpraxisnet.de | https://www.heilpraxisnet.de/naturheilpraxis/ketogene-ernaehrung-kann-ueberlebenszeit-bei-bauchspeicheldruesenkrebs-verdreifachen-20220219554621/ |
| 53 | Does a ketogenic diet help with cancer? | staerkergegenkrebs.de | https://www.staerkergegenkrebs.de/ernaehrung/krebsdiaeten/ketogene-diaet/ |
| 54 | Does a ketogenic diet help with cancer? | krebsinformationsdienst.de | https://www.krebsinformationsdienst.de/leben/alltag/ernaehrung/ernaehrung-diaeten.php |
| 55 | Does a ketogenic diet help with cancer? | blick.ch | https://www.blick.ch/life/gesundheit/medizin/ketogene-ernaehrung-hilft-diese-diaet-gegen-krebs-id6424904.html |
| 56 | Does a ketogenic diet help with cancer? | sueddeutsche.de | https://www.sueddeutsche.de/gesundheit/medizin-aerzte-warnen-krebspatienten-vor-keto-diaeten-1.3687280 |
| 57 | Does a ketogenic diet help with cancer? | christinatomasi.com | https://www.cristinatomasi.com/de/ketose-und-krebs-das-neueste/ |
| 58 | Does a ketogenic diet help with cancer? | zentrum-der-gesundheit.de | https://www.zentrum-der-gesundheit.de/krankheiten/krebserkrankungen/ernaehrungs-tipps-bei-krebs/ernaehrung-krebs |
| 59 | Does a ketogenic diet help with cancer? | gesundheitsstadt-berlin.de | https://www.gesundheitsstadt-berlin.de/ernaehrung-bei-krebs-vorsicht-vor-der-ketogenen-diaet-13626/ |
| 60 | Does a ketogenic diet help with cancer? | spiegel.de | https://www.spiegel.de/gesundheit/diagnose/ketogene-diaet-kann-man-krebs-aushungern-a-1250590.html |
| 61 | Do omega-3 fatty acids prevent cardiovascular diseases? | deutsche-apotheker-zeitung.de | https://www.deutsche-apotheker-zeitung.de/news/artikel/2018/12/18/ema-omega-3-fettsaeuren-nicht-wirksam-zur-sekundaerprophylaxe-nach-herzinfarkt |
| 62 | Do omega-3 fatty acids prevent cardiovascular diseases? | apotheke-adhoc.de | https://www.apotheke-adhoc.de/nachrichten/detail/pharmazie/omega-3-schuetzt-nicht-vor-erneutem-herzinfarkt-sekundaerpraevention/ |
| 63 | Do omega-3 fatty acids prevent cardiovascular diseases? | klartext-nahrungsergaenzung.de | https://www.klartext-nahrungsergaenzung.de/wissen/lebensmittel/nahrungsergaenzungsmittel/omega3fettsaeurekapseln-sinnvolle-nahrungsergaenzung-8585 |
| 64 | Do omega-3 fatty acids prevent cardiovascular diseases? | vitamindoctor.com | https://www.vitamindoctor.com/naehrstoffe/fette-und-fettsaeuren/omega-3-fettsaeuren |
| 65 | Do omega-3 fatty acids prevent cardiovascular diseases? | ndr.de | https://www.ndr.de/ratgeber/gesundheit/Omega-3-Fettsaeuren-fuers-Hers-und-gegen-Entzuendungen,fettsaeuren104.html |
| 66 | Do omega-3 fatty acids prevent cardiovascular diseases? | navigator-medizin.de | https://www.navigator-medizin.de/krankheiten/khk-und-herzinfarkt/koronare-herzkrankheit-khk.html#omega |
| 67 | Do omega-3 fatty acids prevent cardiovascular diseases? | gesundheitsinformation.de | https://www.gesundheitsinformation.de/was-kann-ich-selbst-fuer-meine-herzgesundheit-tun.html |
| 68 | Do omega-3 fatty acids prevent cardiovascular diseases? | lejeune-nh.de | https://www.lejeune-nh.de/omega-3-fettsaeuren-der-schutz-fuer-herz-und-gefaesse/ |
| 69 | Do omega-3 fatty acids prevent cardiovascular diseases? | omega-3-fettsäuren.info | https://xn--omega-3-fettsuren-2qb.info/warum-omega-3-fettsaeuren-fuer-herz-und-kreislauf-wichtig-sind/ |
| 70 | Do omega-3 fatty acids prevent cardiovascular diseases? | quarks.de | https://www.quarks.de/gesundheit/ernaehrung/omega-3-fettsaeuren/ |
| 71 | Does Artemisia help with COVID-19? | medmix.at | https://medmix.at/pflanzliches-in-der-corona-pandemie-beifuss-gegen-coronavirus-erkrankung-covid-19/ |
| 72 | Does Artemisia help with COVID-19? | dw.com | https://www.dw.com/de/artemisia-ein-kraut-gegen-covid-19/a-53936500 |
| 73 | Does Artemisia help with COVID-19? | deutsche-apotheker-zeitung.de | https://www.deutsche-apotheker-zeitung.de/news/artikel/2020/05/11/artemisia-annua-forschung-in-deutschland-ungepruefter-kraeutertee-in-madagaskar |
| 74 | Does Artemisia help with COVID-19? | forumviasanitas.org | https://forumviasanitas.org/thema/artemisia-annua-covid-19/ |
| 75 | Does Artemisia help with COVID-19? | naturundheilen.de | https://www.naturundheilen.de/wissensschatz/tipps-und-erfahrungen/artemisia-annua-gute-wirkung-des-beifuss-extrakts-bei-covid-19-bestaetigt/ |
| 76 | Does Artemisia help with COVID-19? | fu-berlin.de | https://www.fu-berlin.de/presse/informationen/fup/2020/fup_20_107-beifuss-corona/index.html |
| 77 | Does Artemisia help with COVID-19? | deutschlandfunk.de | https://www.deutschlandfunk.de/who-studien-zu-traditioneller-medizin-heilpflanzen-und-corona-100.html |
| 78 | Does Artemisia help with COVID-19? | Wochenblick.at | https://www.wochenblick.at/corona/chinesische-wunder-waffe-gegen-corona-entfernt-spike-protein-aus-zellen/ |
| 79 | Does Artemisia help with COVID-19? | organic-art.eu | https://organic-art.eu/blog/artemisia-annua-wirkung-gegen-corona-covid-19 |
| 80 | Does Artemisia help with COVID-19? | epochtimes.de | https://www.epochtimes.de/gesundheit/medizin/heilkraeuter-gegen-covid-19-studie-bestaetigt-wirksamkeit-a3564289.html |
| 81 | Does saw palmetto help with enlarged prostate? | schwabe.at | https://www.schwabe.at/saegepalme/ |
| 82 | Does saw palmetto help with enlarged prostate? | gesundheitswissen.de | https://www.gesundheitswissen.de/heilpflanzen/heilpflanzen-schmerzen/saegepalme-erfahren-sie-alles-ueber-ihre-heilkraefte/ |
| 83 | Does saw palmetto help with enlarged prostate? | test.de | https://www.test.de/medikamente/wirkstoff/pflanzliches-mittel-saegepalme-w680/ |
| 84 | Does saw palmetto help with enlarged prostate? | deineapotheke.at | https://www.deineapotheke.at/naturheilkunde/heilpflanzen/saegepalme-bei-prostatabeschwerden-3350273 |
| 85 | Does saw palmetto help with enlarged prostate? | phytodoc.de | https://www.phytodoc.de/heilpflanzen/saegepalme |
| 86 | Does saw palmetto help with enlarged prostate? | apotheken-umschau.de | https://www.apotheken-umschau.de/medikamente/heilpflanzen/saegepalme-736547.html |
| 87 | Does saw palmetto help with enlarged prostate? | gutepillen-schlechtepillen.de | https://gutepillen-schlechtepillen.de/saegepalme-keine-hilfe-fuer-prostata/ |
| 88 | Does saw palmetto help with enlarged prostate? | kelterplatz-apotheke.de | https://kelterplatz-apotheke.de/info/news/prostataprobleme-saegepalme-nutzlos-pflanzenextrakte-erzielen-keinen-effekt |
| 89 | Does saw palmetto help with enlarged prostate? | aponet.de | https://www.aponet.de/artikel/saegepalme-9144 |
| 90 | Does saw palmetto help with enlarged prostate? | gesundheitsinformation.de | https://www.gesundheitsinformation.de/was-kann-ich-selbst-gegen-die-beschwerden-tun.html |
| 91 | Do apricot kernels help with cancer? | biokrebs.de | https://www.biokrebs.de/therapien/weitere-therapieansaetze/amygdalin |
| 92 | Do apricot kernels help with cancer? | tumorzentrum-muenchen.de | https://news.tumorzentrum-muenchen.de/2018/07/aprikosenkerne-gegen-krebs-was-ist-dran/ |
| 93 | Do apricot kernels help with cancer? | ugb.de | https://www.ugb.de/exklusiv/fragen-service/schuetzen-aprikosenkerne-vor-krebs/?aprikosenkerne-krebs |
| 94 | Do apricot kernels help with cancer? | verbraucherzentrale.de | https://www.verbraucherzentrale.de/wissen/lebensmittel/nahrungsergaenzungsmittel/amygdalin-b17-64210 |
| 95 | Do apricot kernels help with cancer? | kneipp.ch | https://kneipp.ch/zeitschrift-kneipp/top-themen/bittere-aprikosenkerne-im-kampf-gegen-krebs/ |
| 96 | Do apricot kernels help with cancer? | zentrum-der-gesundheit.de | https://www.zentrum-der-gesundheit.de/ernaehrung/vitamine/b-vitamine/bittere-aprikosenkerne-krebs |
| 97 | Do apricot kernels help with cancer? | krebsinformationsdienst.de | https://www.krebsinformationsdienst.de/aktuelles/2020/news042-aprikosenkerne-krebs-blausaeure.php |
| 98 | Do apricot kernels help with cancer? | gesund.co.at | https://gesund.co.at/bittere-aprikosenkerne-gesund-oder-gefaehrlich-26047/ |
| 99 | Do apricot kernels help with cancer? | naturheilmagazin.de | https://www.naturheilmagazin.de/erkrankungen/krebs/vitamin-b17-bei-krebs/ |
| 100 | Do apricot kernels help with cancer? | dr-baltin.de | https://www.dr-baltin.de/infusionen/vitamin-b17/ |

Note: The URLs were accessed and utilized during November and December 2022. Content at these addresses may have subsequently changed, or the links may no longer be functional.

Abbreviations: COVID-19, coronavirus disease 2019; iWISE, Info without Side Effects

# Final iWISE checklist with explanations for lay users

The English version of the iWISE checklist, including explanations for lay users can also be downloaded here: <https://doi.org/10.48341/iWISE>

# References

1. Viviani M, Pasi G. Credibility in social media: opinions, news, and health information—a survey. WIREs Data Mining and Knowledge Discovery. 2017;7(5):e1209. <https://doi.org/10.1002/widm.1209>.

2. Cambridge Dictionary. Meaning of "layperson" in English: <https://dictionary.cambridge.org/dictionary/english/layperson> (Accessed: 28 March 2022).

3. Fetters MD, Curry LA, Creswell JW. Achieving Integration in Mixed Methods Designs—Principles and Practices. Health Services Research. 2013;48(6pt2):2134-56. <https://doi.org/10.1111/1475-6773.12117>.

4. Griebler U, Kerschner B, Kien C, Klerings I, Lutz B, Krczal E, et al. Development of a quality criteria catalogue for evaluating online health information and the corresponding training interventions for lay people – Study protocol. University for Continuing Education Krems.: <https://osf.io/bfwqh>.

5. O’Brien BC, Harris IB, Beckman TJ, Reed DA, Cook DA. Standards for Reporting Qualitative Research: A Synthesis of Recommendations. Academic Medicine. 2014;89(9):1245-51. <https://doi.org/10.1097/acm.0000000000000388>.

6. American Psychological Association. Mixed Methods Article Reporting Standards (MMARS): <https://apastyle.apa.org/jars/mixed-methods> (Accessed: 27 Nov 2024).

7. Cooper C, Booth A, Husk K, Lovell R, Frost J, Schauberger U, et al. A Tailored Approach: A model for literature searching in complex systematic reviews. Journal of Information Science. 2024;50(4):1030-62. <https://doi.org/10.1177/0165551522111445>.

8. Provost M, Koompalum D, Dong D, Martin BC. The initial development of the WebMedQual scale: Domain assessment of the construct of quality of health web sites. International Journal of Medical Informatics. 2006;75(1):42-57. <https://doi.org/10.1016/j.ijmedinf.2005.07.034>.

9. Powell C. The Delphi technique: myths and realities. J Adv Nurs. 2003;41(4):376-82. <https://doi.org/10.1046/j.1365-2648.2003.02537.x>.

10. Barrett D, Heale R. What are Delphi studies? Evid Based Nurs. 2020;23(3):68-9. <https://doi.org/10.1136/ebnurs-2020-103303>.

11. Jobe JB. Cognitive psychology and self-reports: Models and methods. Quality of Life Research. 2003;12(3):219-27. <https://doi.org/10.1023/A:1023279029852>.

12. Willis GB. Analysis of the cognitive interview in questionnaire design. New York: Oxford University Press; 2015.

13. Scott K, Ummer O, LeFevre AE. The devil is in the detail: reflections on the value and application of cognitive interviewing to strengthen quantitative surveys in global health. Health Policy and Planning. 2021;36(6):982-95. <https://doi.org/10.1093/heapol/czab048>.

14. Beatty PC, Willis GB. Research Synthesis: The Practice of Cognitive Interviewing. Public Opinion Quarterly. 2007;71(2):287-311. <https://doi.org/10.1093/poq/nfm006>.

15. Buers C, Triemstra M, Bloemendal E, Zwijnenberg NC, Hendriks M, Delnoij DMJ. The value of cognitive interviewing for optimizing a patient experience survey. International Journal of Social Research Methodology. 2014;17(4):325-40. <https://doi.org/10.1080/13645579.2012.750830>.

16. Willis G. Pretesting of Health Survey Questionnaires: Cognitive Interviewing, Usability Testing, and Behavior Coding. In: Johnson TP, editor.: Health Survey Methods; 2014. p. 217-42.

17. Patton MQ. Qualitative research & evaluation methods: Integrating theory and practice: Sage publications; 2014.

18. Gale NK, Heath G, Cameron E, Rashid S, Redwood S. Using the framework method for the analysis of qualitative data in multi-disciplinary health research. BMC Medical Research Methodology. 2013;13(1):117. <https://doi.org/10.1186/1471-2288-13-117>.

19. Cochrane Austria. Department for Evidence-Based Medicine and Evaluation. Medizin-Transparent: <https://medizin-transparent.at/> (Accessed: 10 Sep 2024).

20. International Fact-Checking Network (IFCN). Medizin transparent - Universität für Weiterbildung Krems (Donau-Universität Krems): <https://ifcncodeofprinciples.poynter.org/profile/medizin-transparent-universitat-fur-weiterbildung-krems-donau-universitat-krems> (Accessed: 10 Sep 2024).

21. Cochrane Austria. Department for Evidence-Based Medicine and Evaluation. Methodenpapier Medizin-transparent. Version 1.0 vom 5. April 2018: <https://medizin-transparent.at/wp-content/uploads/2022/12/Methodenpapier-Medizin-transparent_v1.0.pdf> (Accessed: 10 Sep 2024).

22. Balshem H, Helfand M, Schünemann HJ, Oxman AD, Kunz R, Brozek J, et al. GRADE guidelines: 3. Rating the quality of evidence. J Clin Epidemiol. 2011;64(4):401-6. <https://doi.org/10.1016/j.jclinepi.2010.07.015>.

23. Santesso N, Glenton C, Dahm P, Garner P, Akl EA, Alper B, et al. GRADE guidelines 26: informative statements to communicate the findings of systematic reviews of interventions. J Clin Epidemiol. 2020;119:126-35. <https://doi.org/10.1016/j.jclinepi.2019.10.014>.

24. Kerschner B, Wipplinger J, Klerings I, Gartlehner G. [How evidence-based are print- and online mass media in Austria? A quantitative analysis] Wie evidenzbasiert berichten Print- und Online-Medien in Österreich? Eine quantitative Analyse. Zeitschrift für Evidenz, Fortbildung und Qualität im Gesundheitswesen. 2015;109(4):341-9. <https://doi.org/10.1016/j.zefq.2015.05.014>.

25. Bürkner P-C. Bayesian Item Response Modeling in R with brms and Stan. Journal of Statistical Software. 2021;100(5):1-54. <https://doi.org/10.18637/jss.v100.i05>.

26. Wickham H, Averick M, Bryan J, Chang W, McGowan LDA, François R, et al. Welcome to the Tidyverse. Journal of open source software. 2019;4(43):1686. <https://doi.org/10.21105/joss.01686>.
